# *CACNA1C* Genetic Variants Differentially Affect Neuronal Networks Through Divergent Pathways

**DOI:** 10.1016/j.bpsgos.2026.100792

**Published:** 2026-07-22

**Authors:** Gemma Wilkinson, Jamie Wood, Nuppu Roivainen, Josephine E. Haddon, Jack F.G. Underwood, Jeremy Hall, Adrian J. Harwood

**Affiliations:** a Neuroscience and Mental Health Innovation Institute, School of Medicine, Cardiff University, Cardiff, United Kingdom; b School of Biosciences, Cardiff University, Cardiff, United Kingdom; c School of Medicine, Cardiff University, Cardiff, United Kingdom; d Department of Psychiatry, Warneford Hospital, University of Oxford, Oxford, United Kingdom; e Mental Health and Neuroscience Institute and Department of Translational Genomics, Maastricht University, Maastricht, the Netherlands

**Keywords:** *CACNA1C*, iPSC, Network activity, Neurodevelopment, Neuron

## Abstract

**Background:**

*CACNA1C* encodes the pore-forming subunit of the L-type calcium channel Cav1.2. Common variants in *CACNA1C* are associated with psychiatric disorders, whereas rare single nucleotide variants cause *CACNA1C*-related disorder, a multisystem disorder with symptoms that include autism spectrum disorder (ASD), intellectual disability, and seizures. However, the cellular mechanisms linking *CACNA1C* dysfunction to neurodevelopmental phenotypes remain poorly understood.

**Methods:**

We generated isogenic *CACNA1C* loss-of-function induced pluripotent stem cell lines and reprogrammed a line from an individual carrying a novel predicted gain-of-function variant (p.Ala1521Pro) in *CACNA1C*. Neuronal activity was assessed using multielectrode arrays, pharmacological manipulation, and gene expression analysis. Early developmental phenotypes were examined using quantitative reverse transcriptase polymerase chain reaction, immunocytochemistry, and RNA sequencing.

**Results:**

Neurons carrying *CACNA1C* variants displayed opposing alterations in network dynamics, depending on variant type. Pharmacological and molecular assays indicated that these network differences were associated with dysregulated GABAergic (gamma-aminobutyric acidergic) signaling. Early developmental analysis revealed that loss of *CACNA1C* altered rosette morphology, CREB (cAMP response element binding protein) phosphorylation, and transcriptional programs related to axonogenesis and synaptic signaling, indicating effects on neuronal differentiation. The patient line exhibited opposing effects on rosette morphology and CREB signaling, reflecting variant-specific effects.

**Conclusions:**

These findings demonstrate that Cav1.2 regulates excitatory-inhibitory balance, network organization, and aspects of neurodevelopment. Divergent effects of *CACNA1C* variants highlight how altered Cav1.2 signaling contributes to variable neurodevelopmental phenotypes, including ASD and epilepsy, and establish a framework for defining *CACNA1C* variant effects in human neurons.

## Plain Language Summary

Changes in *CACNA1C* are linked to neurodevelopmental conditions including autism and epilepsy, but how these changes affect brain cells is not fully understood. We studied human neurons with increased or reduced *CACNA1C* activity and found opposite changes in the way neurons communicate and organize into networks. These differences were linked to altered inhibitory signaling. We also identified changes during early brain cell development, suggesting that *CACNA1C* is important for both brain development and neuronal function.

The L-type calcium channel (LTCC) Cav1.2, encoded by *CACNA1C*, has been strongly associated with psychiatric disorders including bipolar disorder, schizophrenia, and autism spectrum disorder (ASD) via genome-wide association studies (1, 2, 3, 4). Patients with *CACNA1C* variants present with diverse neurodevelopmental symptoms. The most well studied is the gain-of-function (GoF) variant G406R, which causes Timothy syndrome (TS), a multisystem disorder characterized by syndactyly, congenital heart disease, long QT syndrome, developmental delay, ASD, and seizures (5). Patients without the core features of TS, who often carry novel variants, are collectively classified as having *CACNA1C*-related disorder (CRD) (6). They exhibit variable phenotypes; some primarily show cardiac symptoms, whereas others present with neurodevelopmental or neurological features (7,8). To date, only the canonical G406R TS variant has been studied in neuronal models, demonstrating effects on neurogenesis, neuroplasticity, and axonal maturation (9, 10, 11, 12, 13). However, little is known about how different *CACNA1C* variants influence neuronal development and function.

Cav1.2 plays an important role in shaping neuronal activity. It regulates synaptic plasticity via long-term potentiation (LTP), with deletion in rodents leading to reduced LTP and selective memory deficits (14, 15, 16). At the single-cell level, Cav1.2 regulates action potential dynamics; prolonged Cav1.2 opening in TS extends depolarization and widens action potentials (9). At the network level, LTCCs regulate oscillatory activity (17, 18, 19). *CACNA1C* specifically is important in phase coupling of theta and gamma oscillations (16). Finally, in induced pluripotent stem cell (iPSC)–derived neurons, blockade of LTCCs alters synchronized bursting by reducing the interval between bursts (20). Formation of functional neuronal networks is a key aspect of brain development, and synchronized activity is an important intrinsic property of neurons (21, 22, 23, 24, 25). These network-level effects are likely relevant to neuropsychiatric symptoms associated with *CACNA1C*, including ASD and seizures.

Despite clear effects of LTCCs on network activity, many findings come from pharmacological studies, which cannot distinguish between Cav1.2 and Cav1.3 channels. Consequently, the specific contribution of Cav1.2 to neuronal network formation and synchrony has not been directly investigated in human cellular models. Moreover, the mechanisms linking Cav1.2 function to network excitability remain poorly understood. LTCCs also regulate cortical development, and altered progenitor differentiation or excitatory-inhibitory balance may affect network formation (9,26,27). Understanding how Cav1.2 shapes neuronal network activity could reveal critical insights into the neurophysiological basis of *CACNA1C*-related disorders.

To better understand the role of *CACNA1C*, we investigated iPSC-derived neurons from cell lines with a *CACNA1C* loss-of-function (LoF) variant and a patient with a novel *CACNA1C* variant. Using multielectrode array (MEA) recordings and pharmacological manipulation, we demonstrated that *CACNA1C* critically regulates synchronized neuronal network activity. These findings highlight a potential mechanism by which *CACNA1C* variants contribute to neurodevelopmental and neurological symptoms and suggest avenues for therapeutic intervention.

## Methods and Materials

Complete details of experimental procedures are provided in the Supplemental Methods.

### Cell Lines

*CACNA1C* LoF lines were generated via CRISPR-Cas9 targeting exon 2 in an inducible Cas9 iPSC line. A patient with a *CACNA1C* p.Ala1521Pro variant (NP_955630.3) was recruited via the IMAGINE-ID study, and 2 iPSC clones were generated from peripheral blood. Phenotypic features included autism, developmental delay and intellectual disability, borderline cardiac long QT interval, and muscular hypotonia (7). Three control lines were used: an IBJ4 human iPSC line derived from the BJ fibroblast cell line, HPSI1013i-wuye_2, and an inducible Cas9 line, derived from the IBJ4 line.

### Neuronal Differentiation

Differentiation into cortical neurons followed a modified protocol by Chambers *et al.* (28) using dual SMAD inhibition (SB431542, LDN193189) in N2B27 media (20). This protocol produces a population of predominantly glutamatergic neurons with a small proportion of GABAergic (gamma-aminobutyric acidergic) neurons (20). On day 50, neurons were plated onto MEAs for electrophysiological assays and maintained in BrainPhys medium supplemented with B27 + RA, BDNF (brain-derived neurotrophic factor), and ascorbic acid.

### MEA Recordings and Pharmacology

Neuronal activity was recorded using an Axion Maestro on 24-well MEA plate. Spike detection and synchrony analyses were performed using AxiS software and custom scripts. Bicuculline, diltiazem, and diazepam were applied acutely. Wells with <80% electrode coverage were excluded.

### RNA Sequencing

RNA sequencing (RNA-seq) was performed on day-30 neurons (*n* = 3 per genotype). Libraries were prepared and sequenced by Active Motif. Reads were aligned using STAR, and differential expression was analyzed in R (DESeq2, version 1.30.0) with Benjamini-Hochberg correction (adjusted *p* < .01, |log_2_ fold change| ≥ 0.5). Functional enrichment was assessed using fgsea, with clustering of Gene Ontology (GO) terms via GOSemSim. Tissue and disease enrichment analyses used TissueEnrich and DisGeNET, respectively.

### Protein and Immunocytochemical Analysis

Western blotting and immunocytochemistry were performed using standard protocols. Cav1.2 and CREB (cAMP response element binding protein) were analyzed by Western blot using infrared detection. Immunostaining for Pax6, Ki67, and NeuN was performed on fixed cells, imaged using a Leica DMI6000b microscope, and quantified with CellProfiler.

### Quantitative Polymerase Chain Reaction

RNA was extracted using the RNeasy kit, reverse transcribed with the High-Capacity cDNA kit, and analyzed by quantitative polymerase chain reaction using the Pfaffl method, normalized to GAPDH and β-actin.

### Statistical Analysis

Statistical analyses were performed in GraphPad Prism 8 and R (lme4 version 1.1-37). Normality was assessed using the Anderson-Darling test, and 1-way analyses of variance were applied. For MEA data, statistical analyses were performed using linear mixed-effects models. Data are presented as mean ± SEM, with significance thresholds of *p* < .05.

## Results

### LTCC Blockade Reduces Excitability

**Figure 1:**
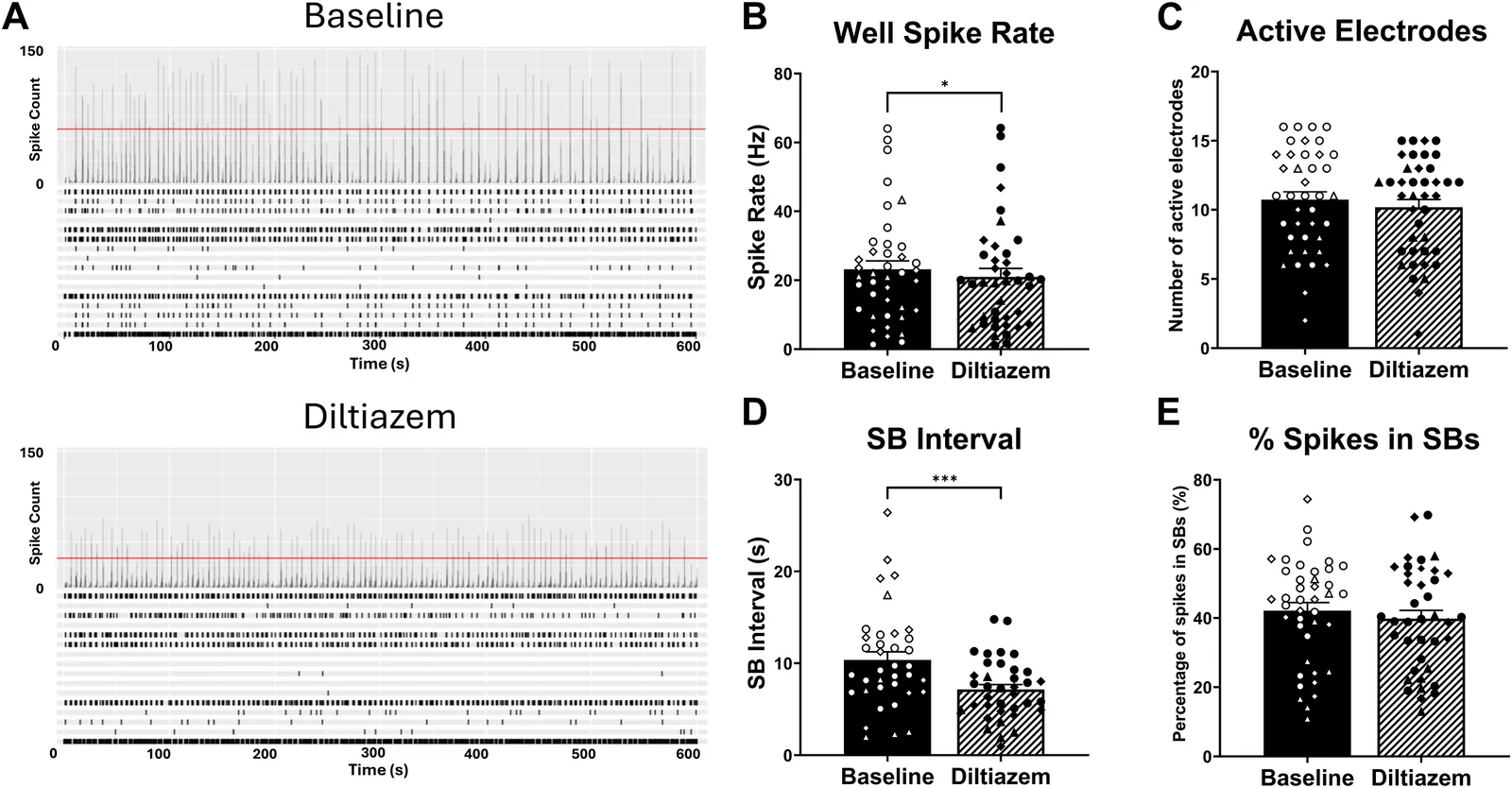
Effect of L-type calcium channel blockade on neuronal network activity. **(A)** Representative raster plots with array-wide spike detection in control neurons at baseline and following 5 μM diltiazem treatment. For both conditions, the upper panel shows the number of spikes per 200-ms bin across a 600-second recording. Vertical scale = 200 spikes per bin. The lower panel shows a raster plot; each row corresponds to an electrode, and spikes are plotted as black lines across the 600-second recording. **(B, C)** Basal excitability is shown by well spike rate and number of active electrodes. **(D, E)** Measures of synchronized activity are mean interval between SBs and percentage of spikes occurring within SBs across the 600-second recording. All plots show mean ± SEM; individual wells are overlaid as points. ∗*p* < .05, ∗∗∗*p* < .001. SB, synchronized burst.

Previous studies have shown that acute blockade of LTCCs alters neuronal network activity (17,18,20). Our initial experiment was aimed at confirming these findings in our model. Control neurons with wild-type *CACNA1C* genes displayed synchronized network activity with repeated periods of bursting behavior (Figure 1A). The LTCC antagonist diltiazem (5 μM) was applied to neurons, and we found a significant reduction in neuronal excitability, demonstrated by decreased well spike rate (Figure 1B) but no significant change in the number of active electrodes (Figure 1C). Consistent with previous studies, diltiazem significantly reduced the interval between synchronized bursts (SBs) (Figure 1D). There was no effect on the percentage of spikes occurring within SBs (Figure 1E). Thus, while burst frequency increased, the distribution of spiking between burst and nonburst periods remained unchanged.

### *CACNA1C* Variants Oppositely Affect Network Activity

To identify the role of *CACNA1C* specifically on neuronal activity, we generated iPSC-derived neurons from a patient carrying a novel variant in *CACNA1C* alongside 2 LoF lines. LoF lines were generated by CRISPR-Cas9–mediated deletions in exon 2 leading to a frameshift and premature stop codon in exon 3 (Figure S1B–E). Amplification of exons 1 to 4 of *CACNA1C* transcript demonstrated no wild-type transcript in mutant lines (Figure S1C). Both lines showed a 75% reduction of detectable Cav1.2 protein (Figure S1F). The functional capacity of the residual protein is unclear. Therefore, while these lines do not represent a complete loss of *CACNA1C*, they model a substantial reduction in Cav1.2 protein levels. The iPSCs derived from our patient line contain a novel single nucleotide variant in *CACNA1C*, p.Ala1521Pro in exon 38 (Figure S1A). Although uncharacterized, this variant is predicted to be deleterious by 2 pathogenicity predictor algorithms, PolyPhen-2 and Provean (29,30). Western blot showed that this variant had no effect on protein expression of Cav1.2 in neurons (Figure S1G). This suggests that phenotypes in this cell line result from altered channel function rather than expression.

**Figure 2:**
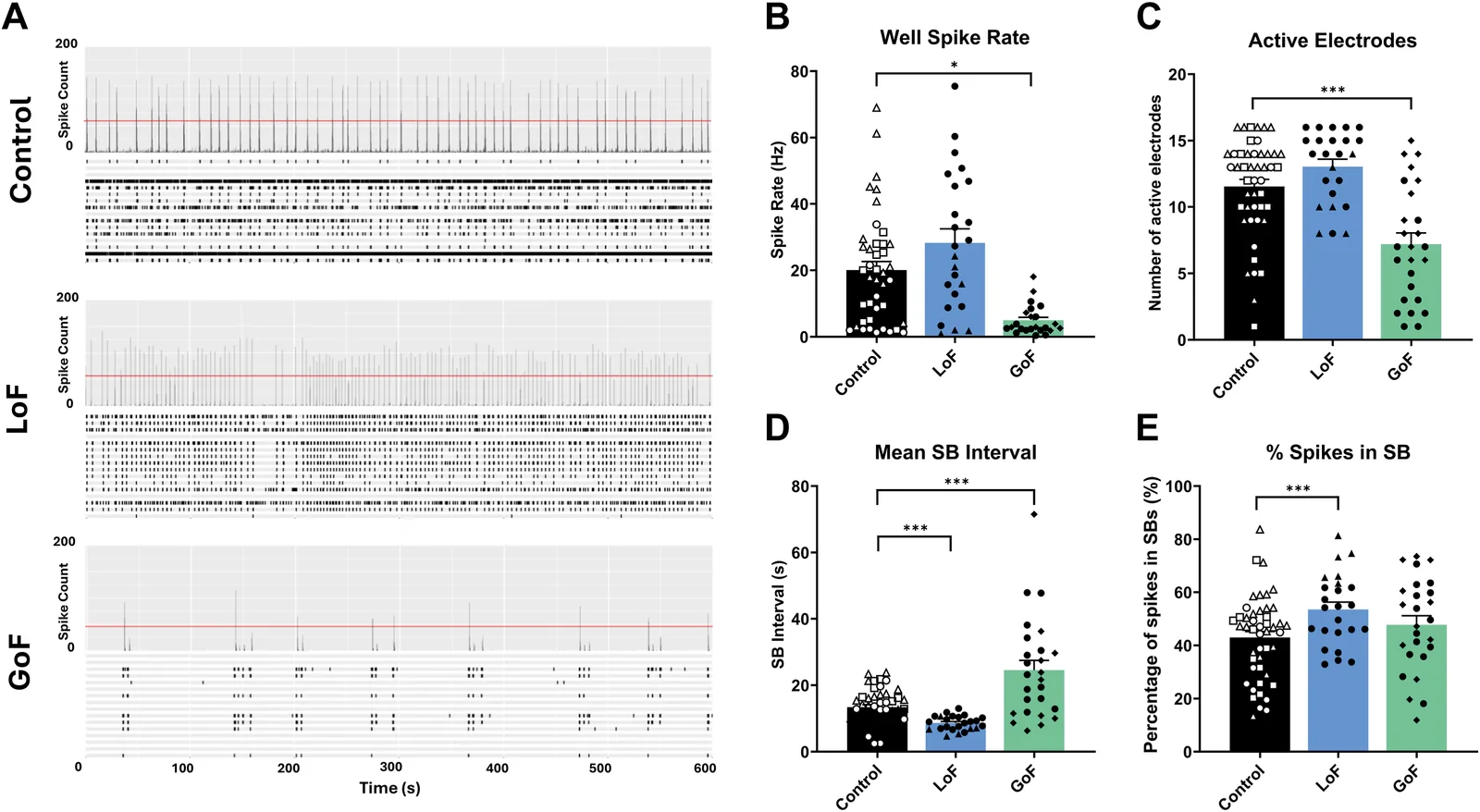
Changes to *CACNA1C* alter neuronal network activity. **(A)** Representative raster plots with array-wide spike detection in control, LoF, and GoF neurons. For each group, the upper panel shows the number of spikes per 200-ms bin across a 600-second recording. Vertical scale = 200 spikes per bin. The lower panel shows a raster plot; each row corresponds to an electrode, and spikes are plotted as black lines across the 600-second recording. **(B, C)** Basal excitability is shown by well spike rate and number of active electrodes. **(D, E)** Measures of synchronized activity are mean interval between SBs and percentage of spikes occurring within SBs across the 600-second recording. All plots show mean ± SEM; individual wells are overlaid as points. ∗*p* < .05, ∗∗∗*p* < .001. GoF, gain-of-function; LoF, loss-of-function; SB, synchronized burst.

Recordings of spontaneous activity using MEAs on day 100 showed LoF, and patient neurons formed synchronized network behavior (Figure 2A). However, they exhibited distinct and opposing effects suggesting a possible GoF effect on Cav1.2 activity in the patient line. Patient (GoF) neurons exhibited a significant reduction in excitability, evidenced by decreased spike rate, and fewer active electrodes (Figure 2B, C). LoF lines showed no significant changes in spike rate or active electrodes compared with controls. LoF and GoF lines exhibited opposing changes in SB interval (Figure 2D). Specifically, LoF lines displayed more frequent SBs with shorter intervals between them, whereas GoF lines had fewer bursts with longer intervals (Table S4). LoF lines showed an increased percentage of spikes occurring within SBs, indicating altered distribution of spiking between burst and nonburst periods (Figure 2E). Analysis of spike waveforms demonstrated no change in spike amplitude in either condition. However, GoF lines displayed a small but significant increase in spike half-width of approximately 12%, with no difference in LoF lines compared with controls (Figure S2A–D).

### GABAergic Signaling Shapes Network Phenotypes

**Figure 3:**
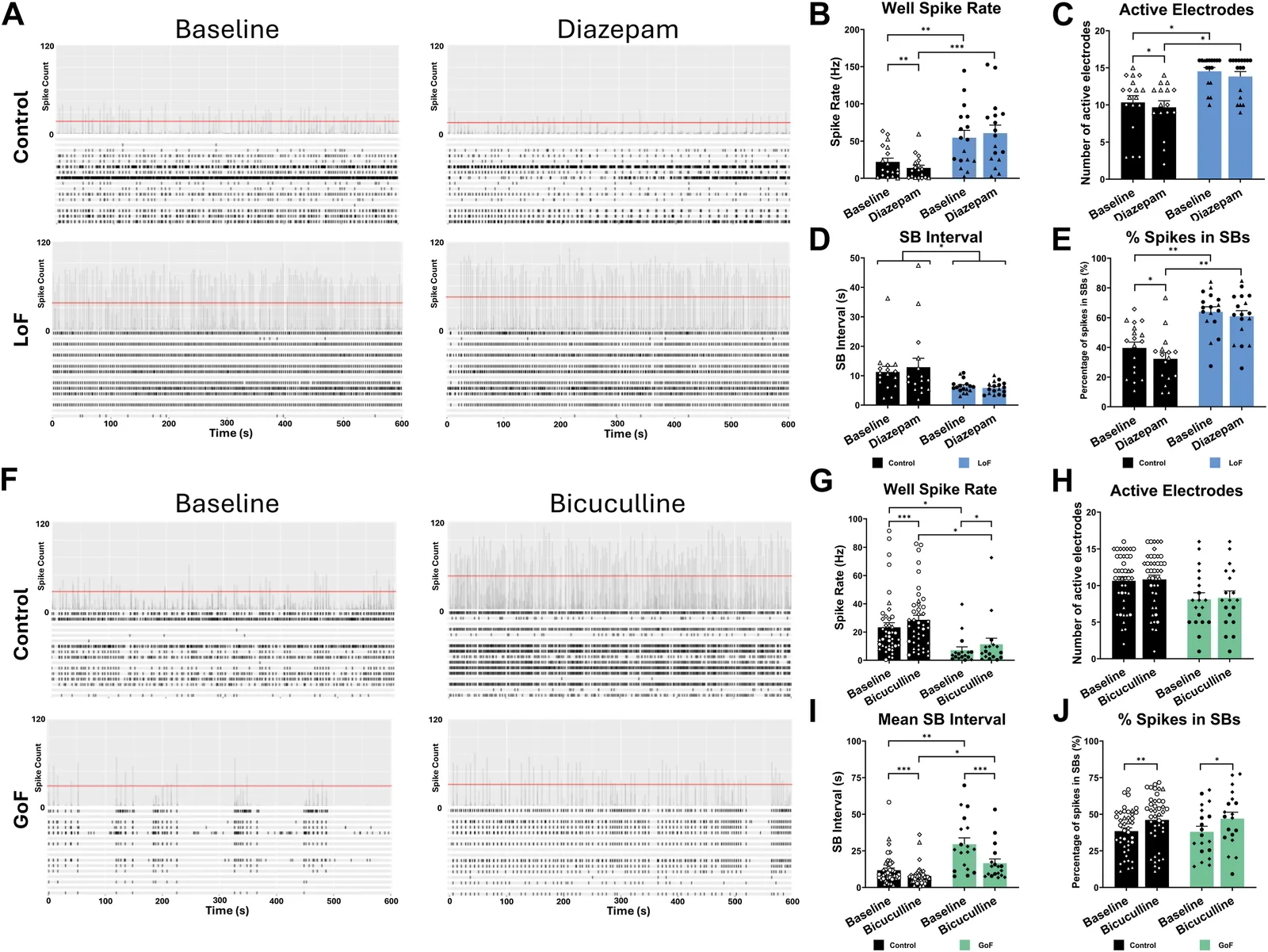
Pharmacological targeting of GABA_A_ receptors in *CACNA1C* LoF and GoF lines. **(A)** Representative raster plots with array-wide spike detection in control and *CACNA1C* LoF lines at baseline and following 50 nM diazepam treatment. For each condition, the upper panel shows the number of spikes per 200-ms bin across a 600-second recording. Vertical scale = 200 spikes per bin. The lower panel shows a raster plot; each row corresponds to an electrode, and spikes are plotted as black lines across the 600-second recording. **(B–E)** Quantification of basal excitability and synchronized activity is shown by well spike rate, number of active electrodes, mean interval between SBs, and percentage of spikes occurring within SBs at baseline and following diazepam treatment in control and *CACNA1C* LoF lines. **(F)** Representative raster plots with array-wide spike detection in control and *CACNA1C* GoF lines at baseline and following 10 μM bicuculline treatment. **(G–J)** Quantification of basal excitability and synchronized activity is shown by well spike rate, number of active electrodes, mean interval between SBs, and percentage of spikes occurring within SBs at baseline and following bicuculline treatment in control and *CACNA1C* GoF lines. All plots show mean ± SEM; individual wells are overlaid as points. ∗*p* < .05, ∗∗*p* < .01, ∗∗∗*p* < .001. GABA, gamma-aminobutyric acid; GoF, gain-of-function; LoF, loss-of-function; SB, synchronized burst.

GABA plays an important role in controlling network activity, including the interval between SBs (31,32). Therefore, we targeted GABAergic signaling to determine whether we could rescue the network changes in the *CACNA1C* mutant lines. Given the opposing activity profiles of the 2 models, pharmacological approaches were selected to bidirectionally modulate network excitability. In the LoF neurons, we applied diazepam (50 nM), a GABA_A_ positive allosteric modulator, to enhance inhibitory signaling and increase the burst interval (Figure 3A) (33). Within the pharmacology experiment, LoF neurons exhibited a higher baseline spike rate than controls, which persisted following diazepam treatment (Figure 3B). Diazepam led to a significant reduction in spike rate in controls but had no effect on LoF neurons. Active electrode number showed a similar pattern and was significantly higher in LoF neurons at both baseline and after diazepam treatment (Figure 3C). Again, diazepam significantly reduced this measure only in controls. Notably, this increase in spike rate and active electrodes in LoF neurons was not observed in the initial baseline comparison of control, LoF, and GoF cultures, indicating that these measures varied across experiments. Diazepam treatment had no significant effect on SB interval in control or LoF neurons (Figure 3D). There was a significant reduction in SB interval in LoF neurons compared with controls, consistent with the previous experiment. However, post hoc comparisons between control and LoF lines within either the baseline or diazepam-treated conditions were not significant. The percentage of spikes in SBs was also elevated in LoF neurons at both time points, but again only controls exhibited a significant reduction in this measure following diazepam (Figure 3E). Overall, diazepam reduced network excitability and the distribution of spiking during burst periods, but only in control lines, suggesting resistance to GABA_A_ receptor modulation in LoF cultures.

In GoF neurons, we applied bicuculline (10 μM), a GABA_A_ receptor antagonist, to reduce inhibitory tone and thus increase excitability and reduce burst interval (Figure 3F) (20). Consistent with previous results, spike rate was significantly reduced in GoF neurons compared with controls at baseline and posttreatment (Figure 3G). Bicuculline application led to a significant increase in spike rate in both control and GoF. Although the number of active electrodes was lower in GoF neurons, it did not reach significance in this experiment, and bicuculline had no significant effect on this measure (Figure 3H). For mean SB interval, GoF lines showed a significant increase at baseline and after treatment (Figure 3I). Bicuculline significantly shortened the SB interval in both controls and GoF lines. Although there were no group differences in the percentage of spikes within SBs at baseline, bicuculline treatment led to a significant increase in the percentage of spikes in SBs in both groups (Figure 3J). Application of bicuculline partially reversed the hypoactive phenotype in GoF neurons, showing a strong effect on synchronized activity.

### Altered Excitatory and Inhibitory Signaling Underlies Network Phenotypes

**Figure 4:**
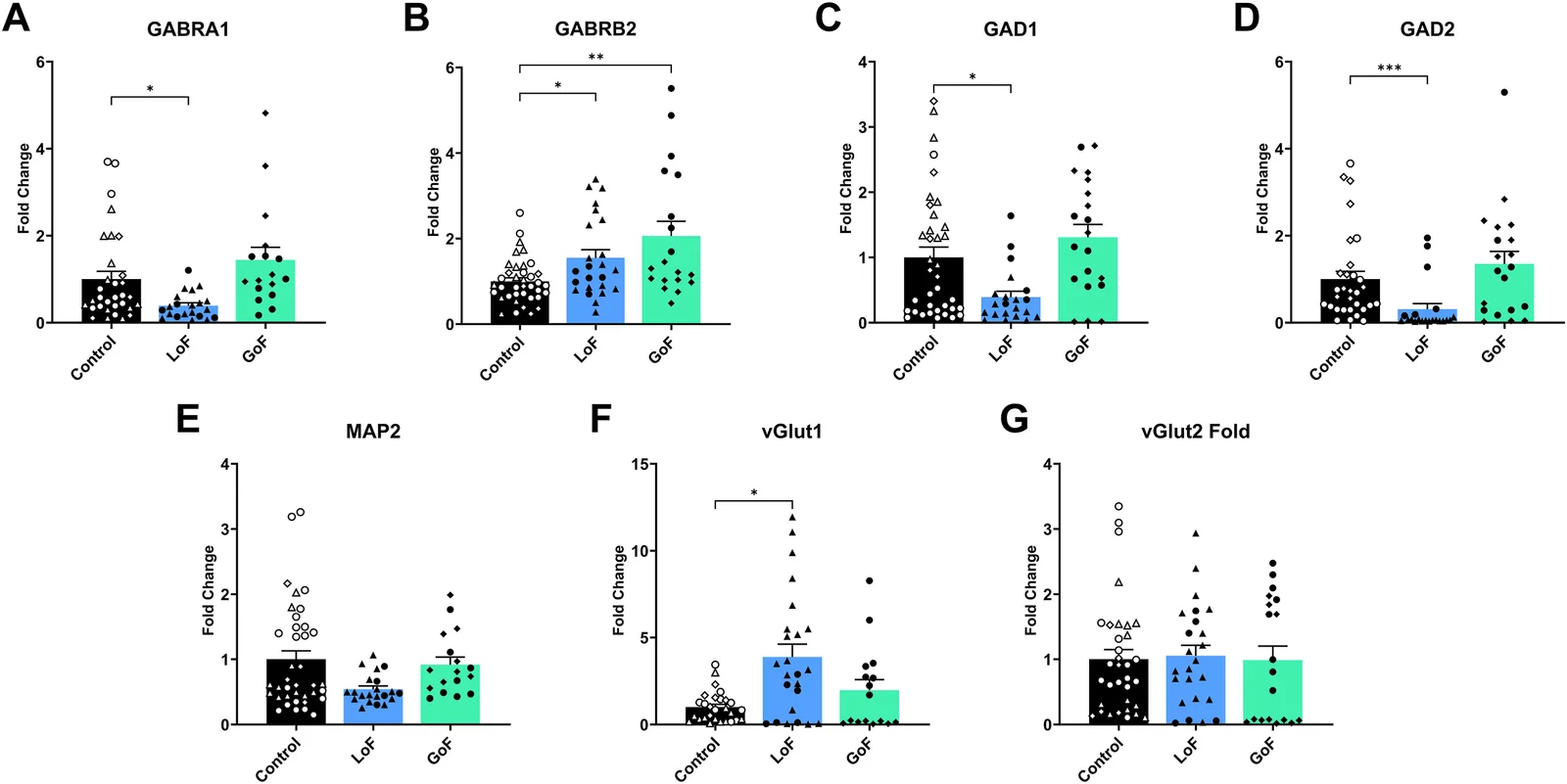
Expression analysis of genes encoding neuronal signaling components in control, *CACNA1C* LoF, and *CACNA1C* GoF neurons on day 120. **(A–D)** Analysis of a panel of inhibitory GABA signaling genes (*GABRA1*, *GABRB2*, *GAD1*, *GAD2*). **(E)** Analysis of neuronal marker *MAP2*. **(F, G)** Analysis of glutamate transporters as markers of excitatory neurons (vGlut1, vGlut2). The values are presented as fold change compared with control lines and the expression of housekeeping genes *GAPDH* and β-actin. All plots show mean ± SEM; individual wells are overlaid as points. ∗*p* < .05, ∗∗*p* < .01, ∗∗∗*p* < .001. GABA, gamma-aminobutyric acid; GoF, gain-of-function; LoF, loss-of-function.

Next, we investigated expression of genes important in GABAergic signaling. *GABRA1* and *GABRB2* encode the alpha1 and beta2 subunits of GABA_A_ receptors and form the most abundant GABA_A_ isoform. *CACNA1C* LoF neurons showed a significant reduction in *GABRA1*, although there was no significant change in GoF neurons (Figure 4A). Both GoF and LoF neurons showed a significant increase in *GABRB2*, with a more pronounced increase in the GoF neurons (Figure 4B). *GAD1* and *GAD2* encode the enzymes responsible for producing GABA. LoF neurons showed a significant reduction in both *GAD1* and *GAD2*, whereas GoF neurons showed no significant changes (Figure 4C, D).

LoF neurons trended toward lower *MAP2* expression (a general neuronal marker), and although the Kruskal-Wallis test indicated a significant group difference, post hoc comparisons with controls were not significant (Figure 4E). Next, we evaluated glutamate transporters vGlut1 and vGlut2 (Figure 4F, G). LoF neurons showed increased vGlut1 expression, while vGlut2 levels remained unchanged across groups, consistent with its lower abundance in mature neurons. Altogether, these transcriptional changes indicate altered expression of genes associated with excitatory and inhibitory signaling, particularly in LoF lines, that may underlie the observed neuronal activity changes and resistance to diazepam.

### Transcriptional Changes Suggest Developmental Contribution

**Figure 5:**
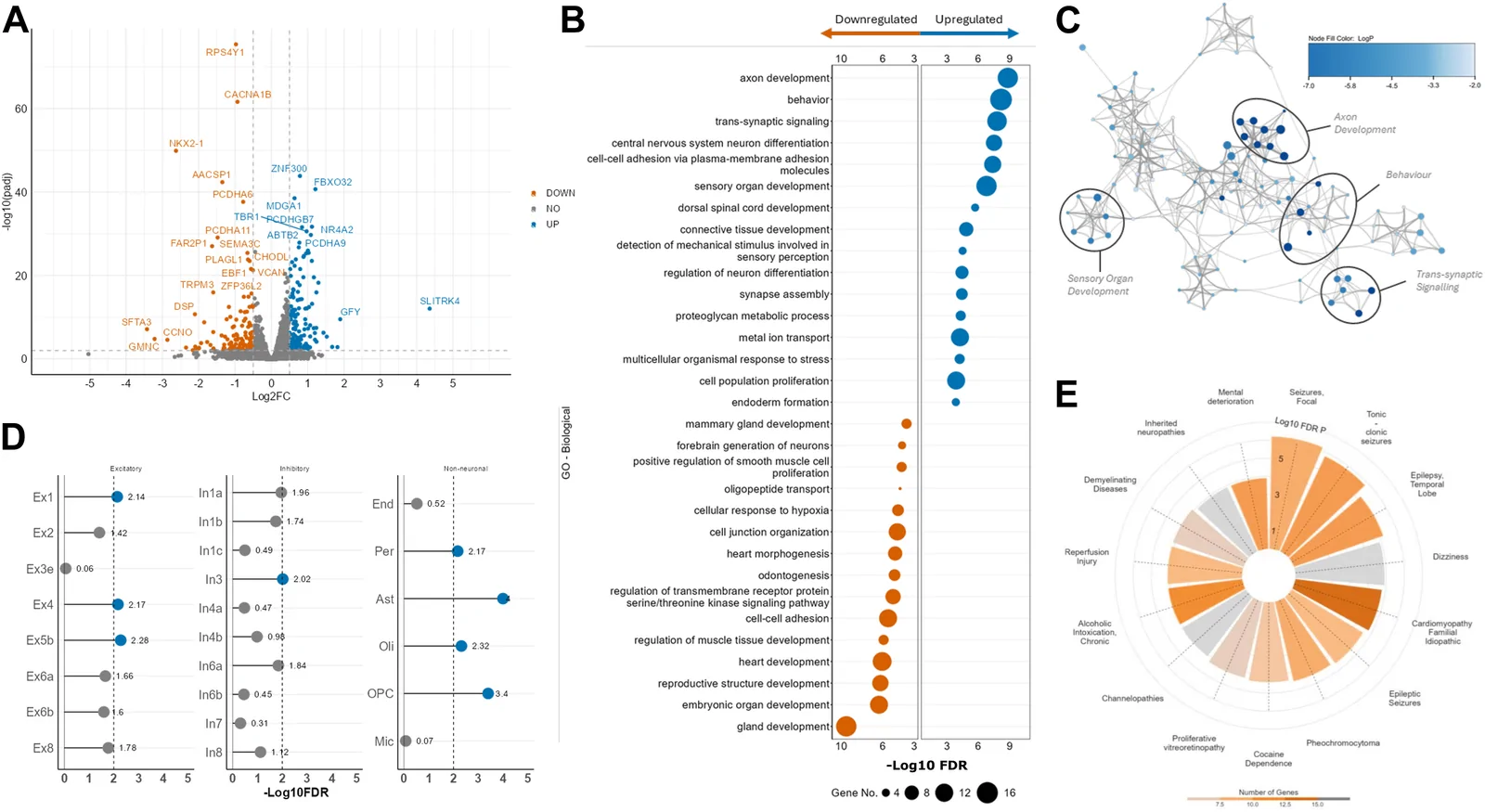
Differential gene expression of *CACNA1C* LoF cell line on day 30 compared with isogenic control cell line. **(A)** RNA sequencing volcano plots of DEGs in a *CACNA1C* LoF mutant compared with wild type, determined by the DESeq2 method with a 0.5-FC threshold and an FDR-adjusted *p* < .01. **(B)** Ranked enrichment analysis of *CACNA1C* LoF gene expression signatures using the GOBP database by the fgsea multilevel enrichment test and genes ranked by minimum significant difference (signed msd) (fgsea). **(C)** Network of enriched GOBP terms, clustered by shared ID and colored by *p* value, for upregulated DEGs. Enriched GOBP terms were clustered based on semantic similarity using GOSemSim (R package), and clusters were manually summarized. Node size indicates the number of genes. **(D)** Brain cell-type enrichment analysis using gene expression per tissue based on brain tissue single-cell data. Significant cell types (*p* < .05) are in blue. **(E)** Disease-gene enrichment analysis performed for all DEGs and disease associations from DisGeNET, with significance determined as *p* < .001. Bar plot color indicates the number of enriched genes within a given disease gene-set. Ast, astrocytes; DEG, differentially expressed gene; End, endothelial cell; FC, fold change; FDR, false discovery rate; GOBP, Gene Ontology biological process; LoF, loss-of-function; Mic, microglia; Oli, oligodendrocytes; OPC, oligodendrocyte precursor cells; Per, pericytes.

To explore the origins of the network phenotype, we performed RNA-seq of day-30 samples in control and LoF cultures corresponding to a period when high neurogenesis had occurred in cultures. Differential expression analysis identified 142 upregulated and 128 downregulated genes compared with control cultures (Figure 5A and Table S5). Upregulated genes were enriched for GO biological processes related to axon development, behavior, trans-synaptic signaling, synapse assembly, and regulation of neuron differentiation (Figure 5B, C and Table S6). In contrast, downregulated genes were enriched for terms associated with forebrain neuron generation, cell junction organization, cell-cell adhesion, and regulation of transmembrane receptor protein serine/threonine kinase signaling.

Cell-type enrichment analysis of these data revealed increased transcription of genes associated with the EX1, 4 and 5b subset of excitatory neurons but no substantial enrichment for inhibitory neurons (Figure 5D). Consistent with this, the interneuron transcription factors *NKX2.1* and *LHX6* were significantly downregulated, alongside *LGI2*, a gene required for inhibitory synapse formation (Table S5). These findings suggest a shift in excitatory- and inhibitory-associated gene expression profiles, consistent with the functional changes observed in MEA experiments. We also observed enrichment of astrocyte and oligodendrocyte gene signatures, further indicating altered cell type–associated transcriptional profiles at this stage. Finally, disease association analysis highlighted strong links between the *CACNA1C* LoF gene signature and epilepsy-related phenotypes. Of the 14 diseases linked to *CACNA1C* loss, 4 were related to epilepsy and seizures, with focal seizures showing the strongest association, consistent with the MEA findings of altered burst dynamics and increased seizure risk in patients with CRD (Figure 5E).

### Early Neural Progenitor Cell Signaling Is Altered by *CACNA1C* Variants

**Figure 6:**
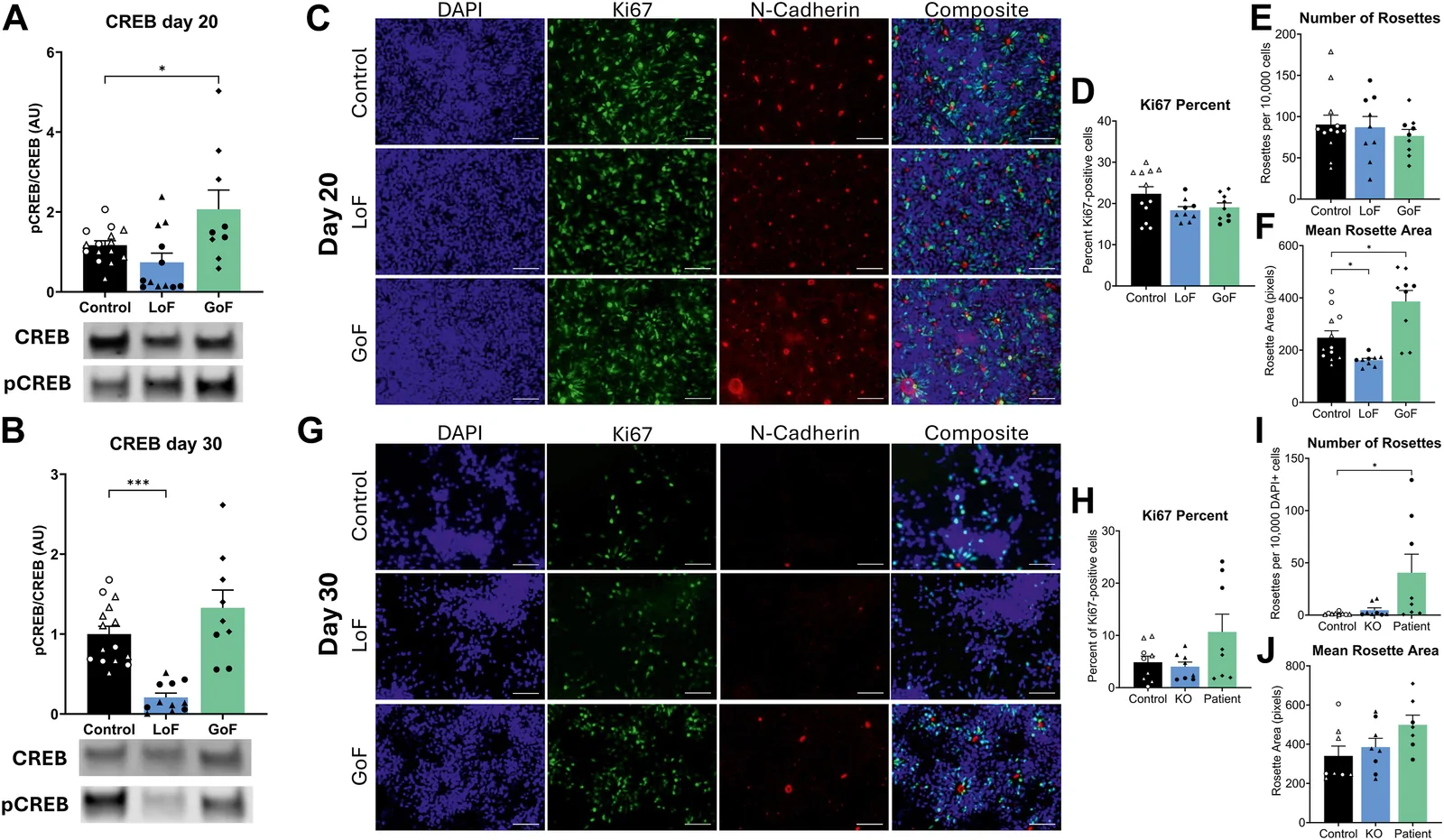
Altered neuronal differentiation of *CACNA1C* LoF and GoF lines. **(A, B)** Phosphorylation of CREB at day 20 and day 30 of differentiation in control, *CACNA1C* LoF, and *CACNA1C* GoF lines. CREB phosphorylation is measured as pCREB divided by total CREB expression. **(C)** Examples images of Ki67 and N-cadherin staining on day 20 of neuronal differentiation in control, *CACNA1C* LoF, and *CACNA1C* GoF lines. **(D)** Quantification of the percentage of DAPI+ nuclei that colocalized with Ki67 on day 20. **(E)** Quantification of number of N-cadherin+ structures normalized to 10,000 DAPI+ nuclei on day 20. **(F)** Mean area of N-cadherin structures on day 20. **(G)** Example images of Ki67 and N-cadherin staining on day 30 of neuronal differentiation in control, *CACNA1C* LoF, and *CACNA1C* GoF lines. **(H)** Quantification of the percentage of DAPI+ nuclei that colocalized with Ki67 on day 30. **(I)** Quantification of number of N-cadherin+ structures normalized to 10,000 DAPI+ nuclei on day 30. **(J)** Mean area of N-cadherin structures on day 30. All plots show mean ± SEM; individual replicates are overlaid as points. ∗*p* < .05, ∗∗∗*p* < .001. CREB, cAMP response element binding protein; GoF, gain-of-function; LoF, loss-of-function; pCREB, phosphorylated CREB.

To determine whether these transcriptional changes may arise due to changes in earlier development, we examined signaling in neural progenitor cells (NPCs) (day 20) and day-30 neuronal cultures, representing a transition from progenitors toward early neuronal differentiation. CREB phosphorylation, a downstream pathway of LTCCs and a regulator of NPC-to-neuron differentiation, showed opposing effects in LoF and GoF lines. On day 20, *CACNA1C* LoF neurons showed no significant change in pCREB (phosphorylated CREB)/CREB but did show a reduction in pCREB/GAPDH and total CREB levels (Figure 6A and Figure S3A, B). In contrast, GoF neurons exhibited increased pCREB/CREB and pCREB/GAPDH without changes in total CREB (Figure 6A and Figure S3A, B). On day 30, reduced CREB signaling persisted in LoF neurons, whereas GoF neurons showed no significant differences in pCREB despite a modest and variable reduction in total CREB (Figure 6B and Figure S3C, D). These divergent changes mirror the opposite effects of LoF and GoF variants on network excitability.

Then we assessed NPC maintenance and rosette organization. There was no difference in expression of Pax6 expression in LoF and GoF lines on day 20 and day 30 (Figure S4A–D). Ki67 expression was also unchanged at both time points (Figure 6C, D, G, H), suggesting no gross differences in progenitor specification or proliferation. In contrast, N-cadherin staining was altered in both LoF and GoF lines at day 20. While there were no changes in the number of rosettes present in cultures, the mean area of rosettes was altered, with LoF lines showing smaller rosettes and GoF lines showing larger ones (Figure 6E, F). Although this difference was lost by day 30 due to overall rosette decline, GoF lines retained more rosettes at this stage (Figure 6I, J), consistent with prolonged NPC maintenance. Because extensive neurogenesis occurs between day 20 and 30, we also quantified NeuN on day 30 as a measure of postmitotic neurons. LoF lines showed a significant decrease in the percentage of NeuN-positive cells, suggesting a reduction in number of neurons, with no significant changes in GoF lines (Figure S4E). The absence of changes in Pax6 or Ki67 at this time point, along with the enrichment of astrocyte and oligodendrocyte genes in the RNA-seq, suggests the emergence of alternative, non-neuronal cell types in LoF cultures. These findings point to distinct effects of *CACNA1C* on neurodevelopment processes, which may contribute to later differences in network activity.

## Discussion

Our results show that *CACNA1C* is a key regulator of neuronal network activity in human iPSC-derived cortical neurons, with opposing effects depending on variant type. These network phenotypes were closely tied to altered GABAergic signaling. Early changes in CREB signaling, NPC maintenance, and neuronal differentiation suggest that developmental mechanisms may contribute to later network differences. These findings highlight how *CACNA1C* may alter network activity through changes in excitatory and inhibitory signaling, potentially contributing to neurodevelopmental phenotypes and susceptibility to disorders such as epilepsy and ASD.

Acute LTCC blockade with diltiazem largely reproduced known effects on network activity, shortening burst intervals as previously reported in iPSC-derived neurons (20). We also observed reduced basal firing, which had not been reported previously in this model. However, LTCC blockade reduced activity in a seizure model, highlighting the role of LTCCs in neuronal firing (34). In contrast, *CACNA1C* LoF neurons exhibited both overlapping and distinct phenotypes; while burst intervals were reduced, LoF lines also showed a higher proportion of spikes occurring within bursts. Although spike rate showed a trend toward increased activity, this did not reach statistical significance in the primary analysis, and in the pharmacological experiments, LoF neurons exhibited a higher baseline spike rate than controls, opposite of the effects of acute LTCC blockade. Similarly, baseline active electrode measures were variable between experiments. This variability may be due to batch effects contributing to a high proportion of the variability seen in MEA experiments (35). Therefore, while network-level measures identified a reproducible LoF phenotype, measures of overall neuronal activity should be interpreted more cautiously. These findings suggest that chronic Cav1.2 loss triggers compensatory or developmental network changes beyond the direct effects of acute LTCC inhibition. Because residual Cav1.2 protein remained detectable in LoF lines, these findings reflect substantially reduced Cav1.2 rather than complete loss of protein. The patient line, carrying a novel A1521P variant, showed an opposite phenotype, with reduced excitability, fewer active electrodes, and prolonged burst intervals, indicating that the variant may have a GoF effect on Cav1.2 channels, although this was not directly assessed in the current study. This was supported by spike waveform features showing increased spike half-width indicating elongation of the action potential. This parallels TS neurons, where prolonged Cav1.2 opening widens action potentials, although effects were subtler in A1521P (9).

Patients with variants in *CACNA1C* show diverse neurodevelopmental symptoms, including developmental delay, intellectual disability, and ASD (7,8). The effect of channel function varies across variants, and our models suggest that both GoF and LoF can perturb development, contributing to these phenotypes. In addition, approximately 40% to 50% of patients develop seizures or epilepsy (7,8). Our findings suggest that LoF may increase seizure susceptibility, whereas the A1521P variant reduced excitability, consistent with the patient’s lack of seizures. In patients, seizures seem to be more common in nontruncating variants of *CACNA1C*, although their effects on channel function vary (8). This suggests that both LoF and GoF may contribute to seizures, and nuances in the effect of each variant influences phenotypic variability. Our patient has an ASD diagnosis, and the neuronal phenotypes are consistent with features previously reported in iPSC-based ASD models showing reduced spontaneous activity, SB frequency, and network complexity (36, 37, 38).

GABAergic signaling emerged as a potential mechanism underlying these network phenotypes. LoF neurons showed reduced GABAergic signaling and diazepam resistance, whereas GoF neurons displayed increased inhibition, as indicated by restored activity after GABA blockade. These results are consistent with prior studies showing that LTCC blockade reduces surface expression of GABA_A_ receptors and decreases the number of parvalbumin interneurons and protein expression of GAD65, GAD67, and VGAT (39, 40, 41, 42). Similarly, heterozygous *CACNA1C* knockout rats show decreased GAD67+ and parvalbumin+ neurons (43). In contrast, LTCC activation leads to increased surface expression of GABA_A_ receptors (44). TS models also show increases in GABA_A_ receptor expression, GAD67, and VGAT puncta (9,45,46). Importantly, dysregulation of inhibitory signaling has been implicated in both ASD and epilepsy. In epilepsy, downregulation of GABAergic signaling leads to disinhibition and hyperexcitability, which can then lead to seizures (47). Both up- and downregulation of GABAergic signaling have been associated with ASD in iPSC-derived models, and our findings reinforce the idea that either increased or decreased GABAergic tone can destabilize developing networks (37,48). Because this protocol predominantly generates glutamatergic neurons, these effects may reflect changes in the small GABAergic subpopulation or broader network-level changes, including GABA_A_ receptor function on excitatory neurons. Future studies using GABAergic-enriched models will help clarify cell type–specific mechanisms.

We also observed developmental alterations that may contribute to later network phenotypes. CRD, ASD, and epilepsy all have strong developmental components, and LTCCs are important in regulation of gene transcription during development. Specifically, Cav1.2 regulates transcription via CREB signaling, and LoF and GoF lines showed opposing effects on CREB phosphorylation. This is consistent with studies showing reduced CREB signaling in Cav1.2 LoF models and increased signaling in TS models (14,16,49). These changes provide a potential mechanism for the changes to GABAergic signaling as LTCC-mediated CREB phosphorylation increases GAD67 levels (41). Cav1.2 channels also activate CamKII (calcium/calmodulin-dependent protein kinase II), upstream of CREB, promoting membrane insertion of GABA_A_ receptors (44). CREB phosphorylation via LTCCs is important for the generation of neurons from NPCs, and CREB is upregulated in NPCs and young neurons (50). This may explain the reduced neuron number and increased glial gene expression in LoF lines. Because this time point precedes robust gliogenesis in dual SMAD inhibition protocols, the transcriptional changes may not reflect mature glial populations but instead indicate altered differentiation trajectories or aberrant gene expression in progenitor populations. This is consistent with rodent studies showing that *CACNA1C*-deficient NPCs generate fewer neurons and more astrocytes, and pharmacological manipulation of LTCCs in NPCs alters neuronal-glial balance (51,52).

Neuron production may also be disrupted by changes to neuronal rosette structure, a critical process in brain development. Rosettes mimic radial organization of the neural tube and are essential for neural patterning and progenitor cell organization. In our model, reduced rosette size in *CACNA1C* LoF lines may reflect impaired cell-cell adhesion. This is consistent with prior evidence that N-cadherin is sensitive to LTCC activity and that loss of Cav1.2 in other systems reduces RhoA/ROCK signaling, a key pathway regulating cadherin-mediated adhesion and rosette integrity during neuronal differentiation (53, 54, 55). Reduced RhoA signaling in *CACNA1C* LoF lines may compromise adherens junction stability, leading to smaller or less organized rosettes, while the inverse may occur in GoF lines. Consistent with this, RNA-seq data indicated broader downregulation of genes involved in cell junction organization, which could affect cortical architecture at later stages. This includes WNT5A, a gene critical for neuronal migration and cortical layering and thus may contribute to altered cortical layer specification previously observed in TS models (9,10,56).

This study highlights the critical role of Cav1.2 in neurodevelopment, demonstrating its impact on excitatory and inhibitory signaling and neuronal network activity. Across models, *CACNA1C* disruption altered CREB signaling, NPC maintenance, and rosette organization, and these early developmental changes may contribute to later shifts in excitatory and inhibitory signaling and network dynamics. Importantly, the *CACNA1C* LoF and GoF lines showed contrasting phenotypes; loss of Cav1.2 reduced network burst intervals, while the A1521P variant led to reduced excitability and prolonged burst intervals. However, these comparisons were not fully isogenic, with LoF and GoF models each representing a single genetic background and only 2 independent control backgrounds (IBJ4/iCas9 and HPSI), so effects of genetic background cannot be excluded; this may underlie the more pronounced effects in the isogenic LoF lines compared with the non-isogenic GoF model. Notably, while robust functional differences were observed in both models, molecular changes in the patient-derived line were more modest and variable, which may reflect its heterozygous state and the lack of an isogenic comparison. Further validation using electrophysiological, calcium imaging, and rescue approaches will be important to directly determine the functional consequences of both the A1521P variant and the residual Cav1.2 protein in the LoF model. Although this work is based on a single patient-derived iPSC line, and we cannot draw conclusions for the wider population of patients with CRD, this model allows investigation of both LoF and GoF effects on human neurobiology. The contrasting phenotypes of LoF and GoF variants underscore how different classes of *CACNA1C* variants may disrupt neuronal development and function through distinct mechanisms. Future studies using multiple patient-derived models with diverse *CACNA1C* variants will be essential to define shared pathways and clarify how specific channel alterations translate into the wide spectrum of neurodevelopmental phenotypes observed in patients. Our work establishes a set of functional readouts, including network activity measures, ERK (extracellular signal-regulated kinase)/CREB pathway activation, and neurodevelopmental patterning phenotypes, providing a valuable framework for evaluating the consequences of *CACNA1C* variants in future studies.

## Supplementary Material

Tables S5–S6

Key Resources Table
