# Supplementary material for: CACNA1C Genetic Variants Differentially Affect Neuronal Networks Through Divergent Pathways
Source: Biol Psychiatry Glob Open Sci. 2026 Jul 22;6(6):100792. doi: 10.1016/j.bpsgos.2026.100792 (PMC13571225; doi:10.1016/j.bpsgos.2026.100792)
Supplement: Supplementary file 1 [file mmc1.pdf]

SUPPLEMENTARY INFORMATION

CACNA1C Genetic Variants Differentially Affect Neuronal Networks Through Divergent Pathways

Wilkinson *et al.*

Supplemental Methods

Human iPSC culture and neuronal differentiation

The cell lines used in this study were human induced pluripotent stem cell lines (iPSCs). The control lines were an IBJ4 human iPSC line derived from the BJ fibroblast cell line (ATCC; CRL-2522), HPSI1013i-wuye_2 (HipSci) and an inducible Cas9 line, which was derived from the IBJ4 line. Cells were cultured on Matrigel (Corning) in Essential 8™ medium (ThermoFisher) at 37°C, 5% CO_2 and passaged with Gentle Cell Dissociation Reagent or Accutase (StemCell Technologies). Differentiation into cortical glutamatergic neurons followed a modified protocol by Chambers et al. using dual SMAD inhibition (SB431542, LDN193189) in N2B27 media (2/3 DMEM/F12; 1/3; neurobasal; B27-RA; N2; 1xPSG; 0.1 mM β-mecaptoethanol)(1,2). Once NPCs were generated cells were cultured in N2B27 with B27 + retinoic acid on PDL (Sigma)/laminin (Roch) at 200000 cells/cm2. At day 50, neurons were plated onto multi-electrode arrays (MEAs) and maintained in BrainPyhs (Stem Cell Technologies) supplemented with B27+RA, PSG; 10ng/ml BDNF and 200µM ascorbic acid. This protocol produces a population of predominately glutamatergic neurons with a small proportion of GABAergic neurons(2).

Generation of *CACNA1C* LoF iPSCs

A CRISPR-Cas9 inducible IBJ4 line (pAAVS1-PDi-CRISPRn; Addgene #73500) was used to target *CACNA1C*. Cas9 expression was induced with doxycycline (2 µg/ml) and cells were transfected with three synthetic gRNAs (5'-CGATGGCCGCCTGCCACGAC-3', 5'-CCCTCCGGATGGGGTTCTTC-3', 5'-TGAATGCCAATGCGGCAGCG-3') using Lipofectamine CRISPRMAX. Successful editing was confirmed by PCR amplification using the flanking primers 5'-GGTGAGGCAAGGAGACTAGAGC-3' and 5'-GATTCATCCAGTCTAGGGCGGG-3'. PCR products were then sequenced using minION long read sequencing (ONT) to identify deletion sizes. Off-target effects were assessed using CRISPOR and RGEN Cas-OFFinder; 12 predicted sites showed no mutations.

Generation of patient CACNA1C iPSCs

A patient with a *CACNA1C* p.Ala1521Pro variant was recruited via the IMAGINE-ID study of individuals with intellectual difficulties of presumed genetic origin (ethics approval: South-East Wales REC). A peripheral whole-blood sample was obtained by JFGU. Full details of the IMAGINE-ID protocol have previously been reported(3). iPSCs were generated from peripheral blood mononuclear cells using the Sendai-based CytoTune-iPS 2.0 kit (A16517, ThermoFisher scientific).

Western blot analysis

Cells were lysed in RIPA buffer (R0278, Sigma) and protease inhibitor cocktail (MSSAFE, Sigma) for 30 mins at 4°C. 20 µg of protein per sample was separated on 4-12% Bis-Tris Plus Gels (Life Technologies) and transferred to nitrocellulose. Membranes were blocked in 5% milk in TBST, probed overnight with primary antibodies. Antibodies used were against Cav1.2 (1:200, Millipore, AB5156), CREB (1:1000, CST, 9104), p-CREB (1:1000, CST, 9198) and GAPDH (1:10,000, Abcam, ab8245). After washing in TBST blots were incubated with IRDye®- conjugated secondary

antibodies (1:10,000, Li-COR). Detection was via Li-COR Odyssey infrared imaging system (Biosciences, Biotechnology). All data normalization was against GAPDH.

Immunostaining Analysis

Cells were fixed in 3.7% PFA, permeabilized (0.3% Triton X-100), blocked (5% donkey serum, 1% BSA), and incubated with primary antibodies overnight at 4°C. Cells were washed with PBS before incubation with secondary antibodies and then counterstained with DAPI (1:2000, Molecular Probes) and mounted in ProLong Gold antifade reagent (Invitrogen). Samples were imaged on a Leica DMI6000b fluorescent microscope. An average of 10 images were taken per coverslip. Primary antibodies were as follows: Pax6 (1:1000, AB_528427, DSHB), Ki67 (1:1000, AB15580, Abcam), N-Cadherin (1:1000, 33-3900, Invitrogen), NeuN (1:1000, ab279297, Abcam). Secondary antibodies were as follows: Alexa Fluor 488-conjugated donkey anti-rabbit (1:500, A32790, Invitrogen), Alexa Fluor 555-conjugated donkey anti-mouse (1:500, A31570, Invitrogen), Alexa Fluor 488-conjugated donkey anti-rat (1:500, A48269TR, Invitrogen). Segmentation and quantification were performed in CellProfiler. Analysis pipelines were developed using a representative random subset of images to define thresholding and object detection parameters, which were then kept constant across all conditions and experiments. Most markers analysed were nuclear (e.g. NeuN, Pax6, Ki67), allowing robust automated segmentation, while membrane-associated structures (N-cadherin) were additionally validated by blinded manual inspection of segmentation outputs.

Bioinformatic Analysis

RNA sequencing was performed on 3 Control and 3 *CACNA1C* LoF samples from iPSC-derived neurons at day 30. Samples were sent to Active motif for library preparation and sequencing. Sequence reads were aligned to the reference genome using the STAR algorithm. Counts were read into R 4.3.2 (https://www.R-project.org/) and filtered to retain genes with counts greater than 5. One *CACNA1C* LoF sample was deemed an outlier by RUVseq and excluded from analysis. Differential gene expression (DGE) analysis was performed using DESeq2 (R package, v1.30.0). P values were adjusted for multiple comparisons using Benjamini-Hochberg correction to control the false discovery rate (FDR). Genes were considered differentially expressed if it has an adjusted P-value of less than 0.01 and a Log2 fold change of ±0.5 or greater.

Enrichment analysis was performed to determine Gene Ontology – Biological Processes (GOBP), using the fgsea multilevel enrichment test and genes were ranked by minimum significant difference signed (msd). Enriched terms with a padj value less than 0.01 were considered significant. Enriched GOBP terms were clustered based on semantic similarity using GOSemSim (R package, v2.24.0), and clusters were manually summarized. Tissue enrichment analysis was conducted using the TissueEnrich (R package, v1.20.0). Disease-gene network analysis was performed using the DisGeNET database (v7.0), calculating overlap significance for DEGs and disease genesets by hypergeometric probability, with adjusted $P<0.001$ considered statistically significant.

Gene Expression Analysis

RNA was extracted from cells using the RNeasy mini kit (Qiagen, Germany). For each sample 500ng of RNA was reverse transcribed using the High-Capacity RT cDNA Kit (ThermoFisher

Scientific). qRT-PCR analysis of mRNA used the qPCRBIO SyGreen Blue Mix (PCR Biosystems, UK). All qRT-PCR reactions were performed in triplicate on a QuantStudio 7 Flex RT-PCR machine (ThermoFisher Scientific) and relative expression was calculated using the Pfaffl method with data normalized to GAPDH and B-Actin (see Table S3 for primer sequences).

MEA and pharmacological manipulation

Day 50 neurons were plated as high density drop cultures (15,000 cells/μl) containing 10 μg/ml laminin onto PEI-treated 24-well CytoView MEAs (M384-tMEA-24 W, 4X4 electrode grid). Cells were cultured in 1:1 fresh to normal human astrocytes (NHA) conditioned BrainPhys media with half-media changes twice a week. Neurons were treated with DAPT (10 μM) for 1 week to remove NPCs and allow synchronized networks to form. This MEA culture and recording pipeline has been previously established in our laboratory(2,4). Activity was recorded using Axion Maestro Pro. Channels were sampled with a gain of 1000 × and a sampling rate of 12.5 kHz/channel. During the recording, the temperature was maintained at 37 °C. Cultures were inspected before recordings and wells with extreme clustering or coverage of less than 80% of electrodes were excluded from analysis. Pharmacological agents were applied in BrainPhys prior to recording. Bicuculline and diazepam were applied for 10 min and 1 h, respectively, before recording. Diazepam required longer incubation to allow for slower network-level modulation consistent with previous studies and its action as a GABAA receptor positive allosteric modulator(5). Diltiazem was applied for 10 minutes to control lines to assess its effects on network activity. After recordings, medium was removed from the MEAs and cultures were washed 3 times with PBS before fresh medium was added.

Spike detection was carried out using AxiS software (Adaptive Threshold Crossing Method, 6 x Standard Deviations). Offline analysis was achieved with custom scripts written in R. Spike timestamps were analyzed to provide statistics on the general excitability of cultures. Network activity was analyzed using array–wide spike detection rate (ASDR) with a bin width of 200 ms. Synchronized bursts were detected from binned data by a 3-step process: The start of a synchronized burst was detected by a spike count of at least 40% of the maximum ASDR. If the subsequent bin(s) also contained a spike count above the threshold it was included in the synchronized burst. The end of a synchronized burst was determined by a period of 400ms or more without a spike count about the threshold.

Spike waveforms were analyzed using custom scripts in MATLAB and R. For each detected spike, aligned samples was extracted and subjected to singular value decomposition (SVD). Spikes were clustered using principal component analysis to classify waveform polarity and morphology. Only unipolar (positive or negative) spikes were retained for quantitative analysis. Spikes were filtered using median absolute deviation (threshold = 4) to remove outliers. Spike half-width at half-maximum (FWHM) was calculated as the temporal interval between half-maximum crossings of the peak amplitude. Axion-reported spike amplitudes were used and defined as the peak voltage of each waveform. Spikes were separated into positive and negative waveforms, and a random sample was selected from each group for visualization.

Statistical Analysis

Data was analyzed in GraphPad Prism 8 and R (lme4 v1.1-37, glmmTMB v 1.1.14). Normality was assessed via Anderson-Darling test; ROUT (Q=1%) was used to exclude outliers and One-way ANOVA was applied. For MEA data, statistical analyses were performed using linear mixed-effects models, with cell line as a fixed effect and differentiation batch as a random effect. Normality of residuals was assessed using Q–Q plots, and non-normally distributed data were log-transformed where appropriate prior to analysis. For pharmacological experiments, where measurements were taken from the same wells before and after treatment, we included well as a random effect to account for repeated measures. As the active electrode measure is a bounded count it was analysed using a beta-binomial mixed effect model. Data are presented as mean and standard error of the mean (SEM). Statistical significance was considered as P-values <0.05, and significances represented as $*P < 0.05$, $**P < 0.01$ and $***P < 0.001$.

Supplemental Tables

Group	Cell line	Genetic background	Genetic modification
Control	IBJ4	IBJ4	None
Control	iCas9	IBJ4	Tet-inducible Cas9 knock-in (AAVS1 locus)
Control	HPSI1013i-wuye_2	Independent (HipSci)	None
CACNA1C LoF	Clone 20	IBJ4 (via iCas9)	CRISPR-induced deletion
CACNA1C LoF	Clone 29	IBJ4 (via iCas9)	CRISPR-induced deletion
CACNA1C A1521P	IM291 clone 24	Patient-derived (IM291)	Endogenous A1521P variant
CACNA1C A1521P	IM291 clone 82	Patient-derived (IM291)	Endogenous A1521P variant

Table S1: Summary of cell lines used in this study. Cell lines are shown for each Group and include information on genetic background and genetic modifications.

ASSAY	Cell Line							No. of differentiations
	Ctrl: IBJ4	Ctrl: iCas9	Ctrl: HPSI1013i-wuye_2	CACNA1C LOF: Clone 20	CACNA1C LOF: Clone 29	CACNA1C A1521P: IM291 C24	CACNA1C A1521P: IM291 C82	
MEA baseline measures	x	x	x	x	x	x	x	3
MEA pharmacology - diltiazem	x	x	x					3
MEA pharmacology - bicuculline	x	x	x			x	x	3
MEA pharmacology - diazepam	x		x	x	x			2
qRT-PCR	x	x	x	x	x	x	x	3
RNA-seq		x			x			3
ICC	x	x		x	x	x	x	3
Western Blot	x	x		x	x	x	x	3

Table S2: Cell Line usage across experiments. Table showing which cell lines were used for each experiment, along with the number of independent differentiations performed.

Gene	Forward	Reverse
GAPDH	AGGCTGGGGCTCATTTG	CAGTTGGTGGTGCAGGAG
B-Actin	TCACCACCACGGCCGAGC	TCTCCTTCTGCATCCTGTCG
GABRA1	GGATTGGGAGAGCGTGTAACC	TGAAACGGGTCCGAAACTG
GABRB2	TCTCTCTGTATACGATGGACCC	GCTTCTGGGGTCTCCAAGTC
GAD65	GGCTTTTGGTCTTTCGGGTC	GCACAGTTTGTTTCCGATGCC
GAD67	GCCAGACAAGCAGTATGATGT	CCAGTTCCAGGCATTTGTTGAT
MAP2	CTGCTTTACAGGGTAGCACAA	TTGAGTATGGCAAACGGTCTG
vGlut1	CGACGACAGCCTTTTGTGGT	GCCGTAGACGTAGAAAACAGAG
vGlut2	GGGAGACAATCGAGCTGACG	CAGCGGATACCGAAGGAGATG
CACNA1C (Exon 2)	GGTGAGGCAAGGAGACTAGAGC	GATTCATCCAGTCTAGGGCGGG
CACNA1C (Exons 1-4)	CATTTCTTCCTCTTCGTGGCTGC	TAGTAGGTTCCAGCCGTTGC

Table S3: Primers for qRT-PCR analysis. This table lists the forward and reverse primer sequences used for qRT-PCR analysis for each gene examined.

Measure	Comparison	Estimate	SE	Statistic	df	p value	Sig
Well Spike Rate (Hz)†	Control vs LoF	0.734	0.533	1.376	59.052	0.1741	
	Control vs GoF	-1.401	0.521	-2.687	35.820	0.0109	*
Median Electrode Spike Rate (Hz)†	Control vs LoF	0.746	0.415	1.796	66.712	0.0770	
	Control vs GoF	-0.191	0.380	-0.503	61.954	0.6169	
Number of Active Electrodes	Control vs LoF	-0.595	0.606	-0.981	NA	0.3268	
	Control vs GoF	-2.720	0.813	-3.344	NA	0.0008	***
Mean SB Interval (s)†	Control vs LoF	1.298	0.270	4.815	82.819	6.56E-06	***
	Control vs GoF	-1.140	0.297	-3.843	84.530	0.0002	***
SB Interval Co-efficient of variation†	Control vs LoF	-0.274	0.145	-1.896	86.889	0.0613	
	Control vs GoF	-0.578	0.134	-4.328	86.802	4.02E-05	***
Number of Synchronized Bursts†	Control vs LoF	0.691	0.294	2.350	44.011	0.0233	*
	Control vs GoF	-1.232	0.251	-4.918	54.512	8.41E-06	***
Mean Spikes per SB†	Control vs LoF	-0.286	0.525	-0.545	22.052	0.5911	
	Control vs GoF	-0.609	0.465	-1.308	11.367	0.2166	
Percent of spikes in SBs (%)	Control vs LoF	31.081	7.106	4.374	90.000	3.27E-05	***
	Control vs GoF	7.736	5.545	1.395	90.000	0.1664	
Mean Spike Amplitude (mV)	Control vs LoF	0.000	0.000	1.589	26.673	0.1238	
	Control vs GoF	0.000	0.000	-0.428	17.476	0.6739	
Mean Spike Half-width	Control vs LoF	0.032	0.016	1.978	76.346	0.0516	
	Control vs GoF	0.052	0.021	2.503	55.477	0.0153	*

Table S4: Summary of statistical analysis of multielectrode array (MEA) measures. Results from linear mixed-effects models (LMM) and beta-binomial mixed models (BBMM) are shown. Estimate represents the model coefficient and SE the standard error of the estimate. Statistic corresponds to the test statistic (t for LMM; Wald z for BBMM), with degrees of freedom (df) reported for LMM only. Associated p values are shown, with statistical significance indicated in the Sig column. Measures marked with † were log-transformed prior to analysis following assessment of normality using Q–Q plots.

Supplementary Tables S5-S6 are provided as separate Excel files due to size.

Table S5: Differential gene expression analysis. DESeq2 results of RNA seq analysis on Control and CACNA1C LoF iPSC-derived neurons at day 30. Columns include gene symbol, baseMean expression, log2 fold change, standard error (lfcSE), Wald statistic, raw p value, and Benjamini–Hochberg adjusted p value (padj).

Table S6: Gene Ontology biological process (GOBP) enrichment analysis. Gene Ontology enrichment results for differentially expressed genes from control and CACNA1C LoF neurons at day 30. Columns include regulation direction, GO term ID, category, term description, gene count, percentage of genes, false discovery rate (FDR), –log10(q), and associated genes.

Supplemental Figures

Figure S1

A

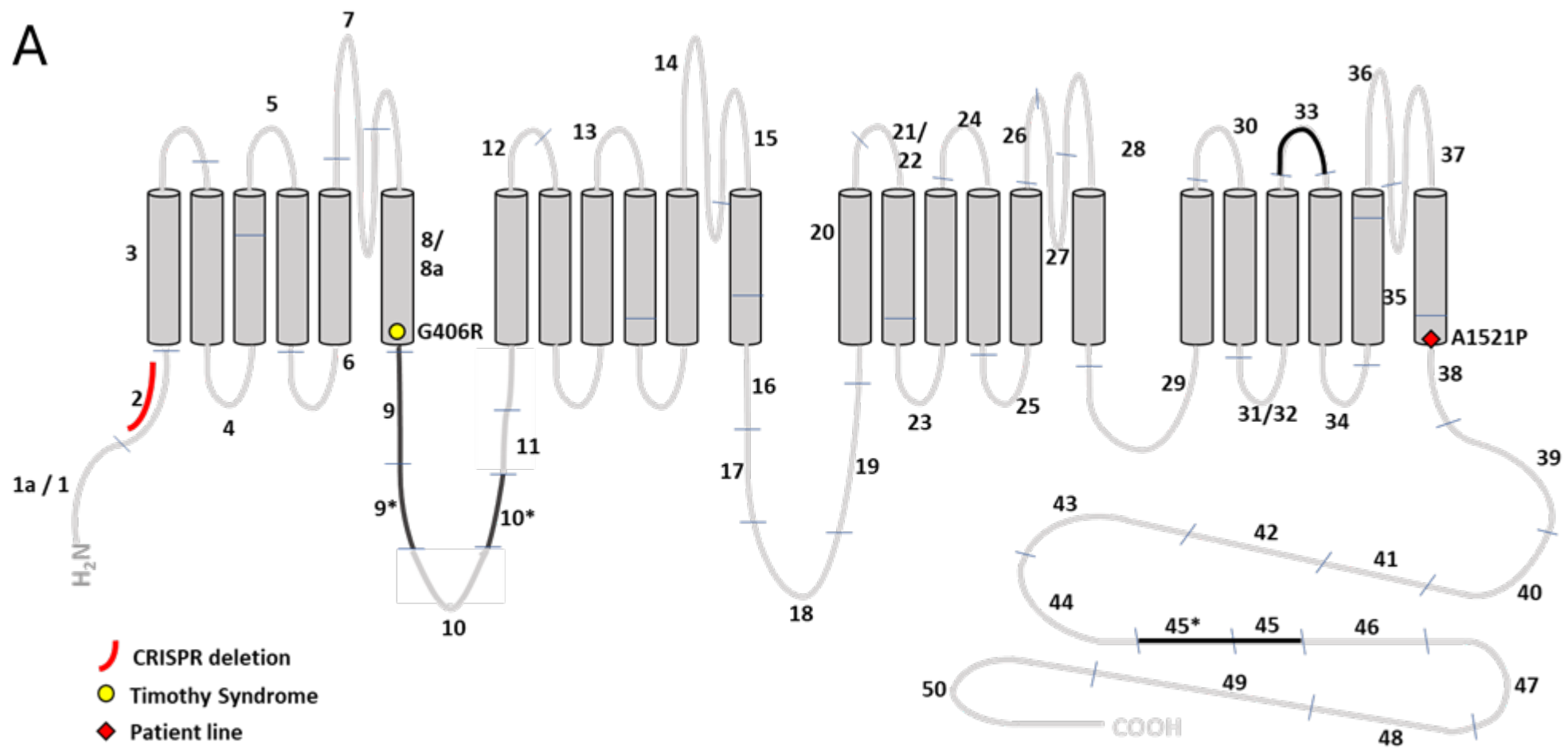

B

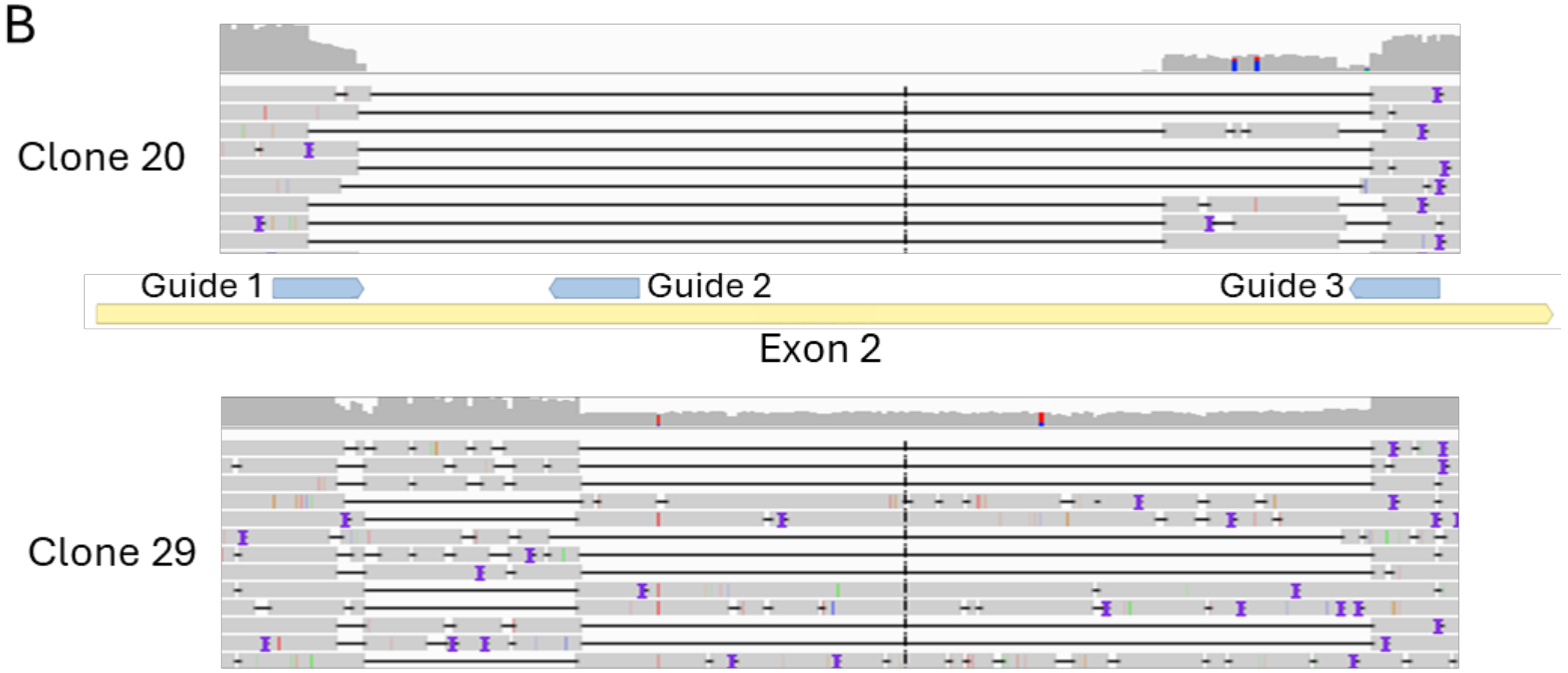

C

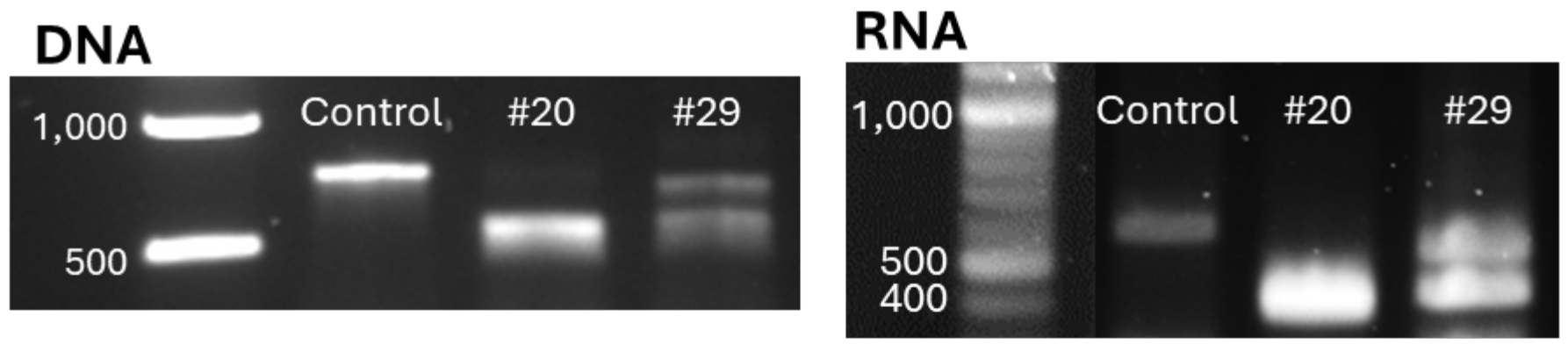

D

Clone	INDELs	
20	224 bp deletion	183 + 8bp deletion
29	6 bp + 176 bp deletion	47 bp deletion

E

Wild-type

GSNYGSPRPAHANM ... PIRRACISIVEWKPFEIIILLT...

Deletions

GSNYGSPRPAHANM ... PHPEGLHQHCRMETI *

F

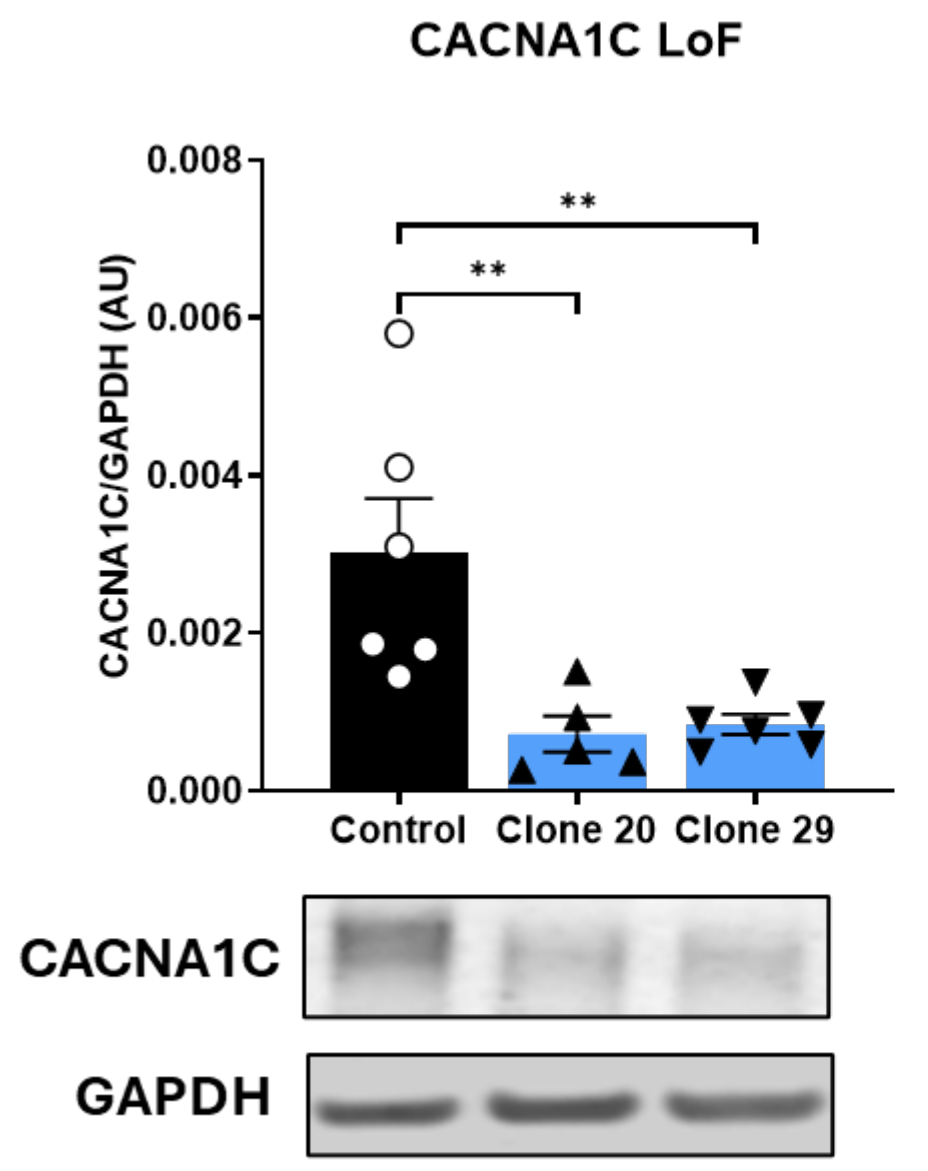

G

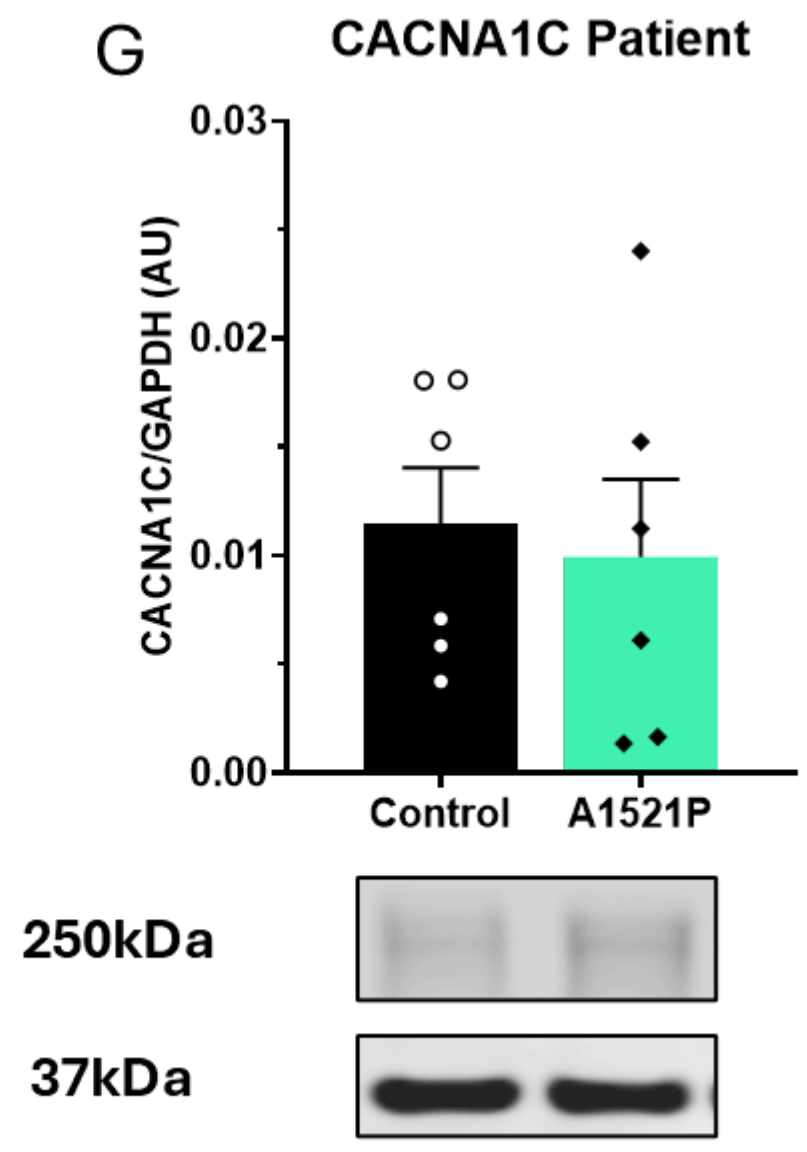

Figure S1: Generation of CACNA1C LoF lines and validation of protein expression in CACNA1C LoF and Patient A1521P lines A: Diagram of CACNA1C structure showing location of typical Timothy Syndrome mutation, deletion in LoF lines and point mutation in patient line. **B:** Sequencing results of the two clones that contained indels. Each row is an individual read from the sequencing and the black lines denote deletions when compared to the reference genome. The position of the guides on exon 2 is shown and is the same scale as the sequencing data. **C:** PCR product of DNA and RNA transcript for target region in iCas9 iPSCs (control) and the two clones that contained indels (20 and 29). **D:** Summary of deletion sizes present in clones. **E:** Deletions in exon 2 of CACNA1C LoF lines leads to introduction of stop codon in exon 3 of CACNA1C. **F:** Western Blot of CACNA1C with GAPDH as housekeeping in iCas9 and CACNA1C LoF lines at day 50 of neuronal differentiation **G:** Western Blot of CACNA1C with GAPDH as housekeeping in control and patient A1521P lines at day 50 of neuronal differentiation. Plots show mean ± SEM, individual samples are overlayed as points. *p < 0.05, **p < 0.01, *** p < 0.001, **** p < 0.0001.

Figure S2:

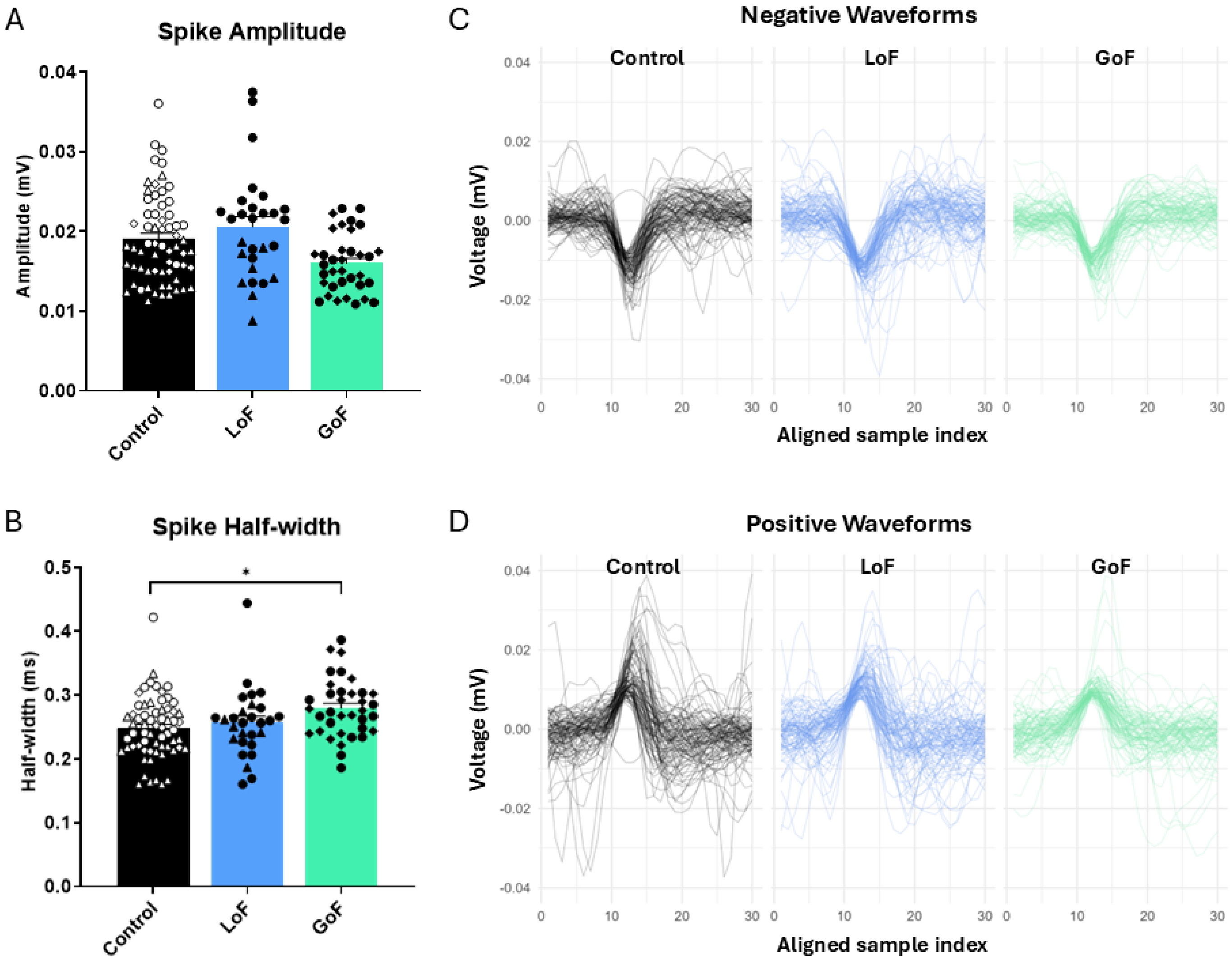

A
Spike Amplitude
Amplitude (mV)
0.04
0.03
0.02
0.01
0.00
Control
LoF
GoF
B
Spike Half-width
Half-width (ms)
0.5
0.4
0.3
0.2
0.1
0.0
*
Control
LoF
GoF
C
Negative Waveforms
Control
LoF
GoF
Voltage (mV)
0.04
0.02
0.00
-0.02
-0.04
0
10
20
30
Aligned sample index
D
Positive Waveforms
Control
LoF
GoF
Voltage (mV)
0.04
0.02
0.00
-0.02
-0.04
0
10
20
30
Aligned sample index

Figure S2: Spike waveform features in control, CACNA1C LoF and CACNA1C GoF neurons. A: Mean amplitude of spikes during MEA recordings. **B:** Mean half-width of spikes during MEA recordings. **C-D:** Examples of positive and negative unipolar waveforms used for analysis of spike features. A random selection 100 waveforms were used for each condition. Bar plots show mean ± SEM, individual replicates are overlayed as points and are representative of well-averages. $*p < 0.05$, $**p < 0.01$, $***\ p < 0.001$.

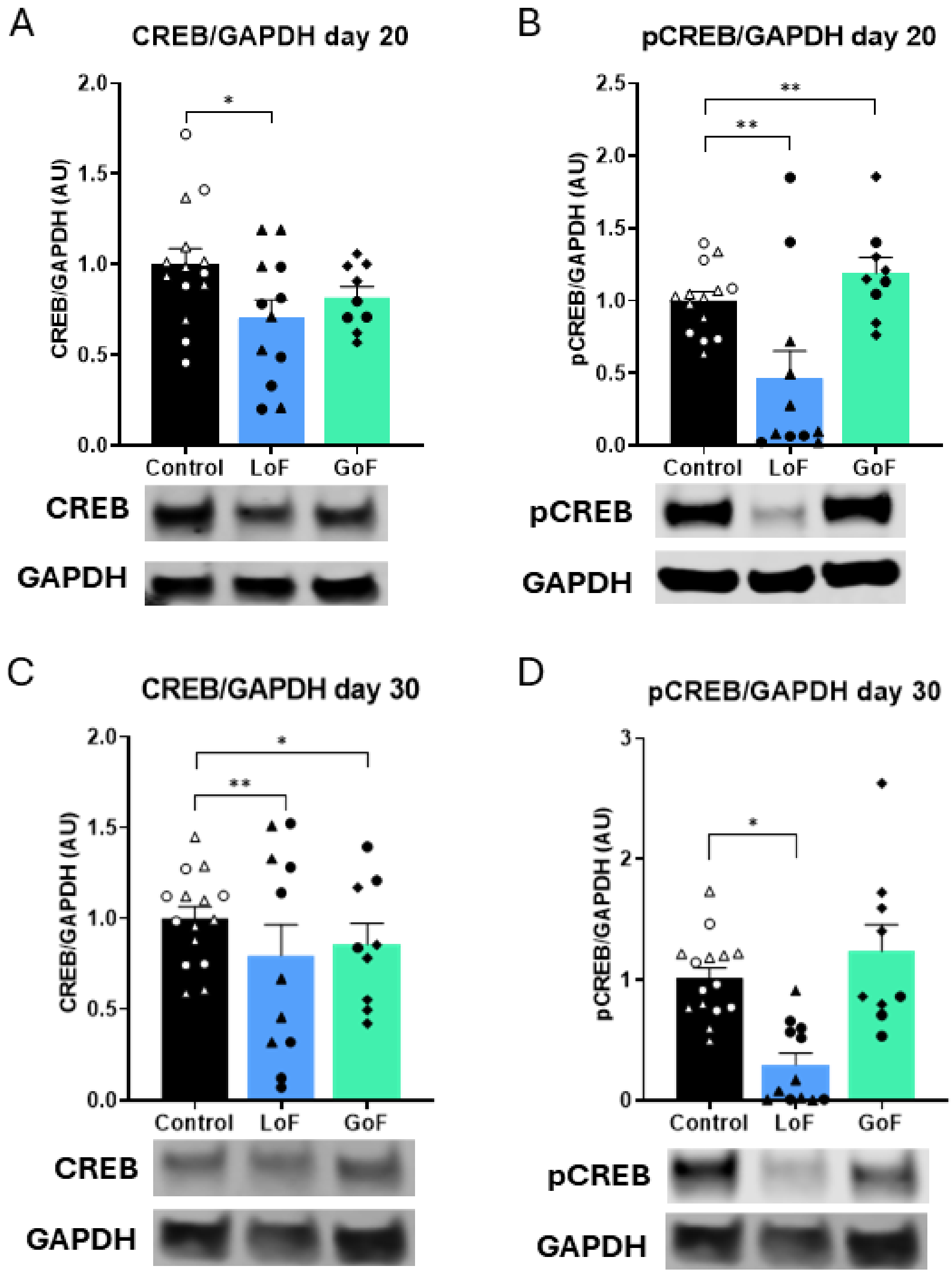

Figure S3: Normalized CREB protein expression in control, CACNA1C LoF and CACNA1C GoF lines. A: CREB relative to housekeeping gene GAPDH at day 20. B: pCREB relative to GAPDH at day 20. (C) CREB relative to GAPDH at day 30 (D) pCREB relative to GAPDH at day 30. Plots show mean ± SEM, individual replicates are overlayed as points. $*p < 0.05$, $**p < 0.01$, $***\ p < 0.001$.

Figure S4:

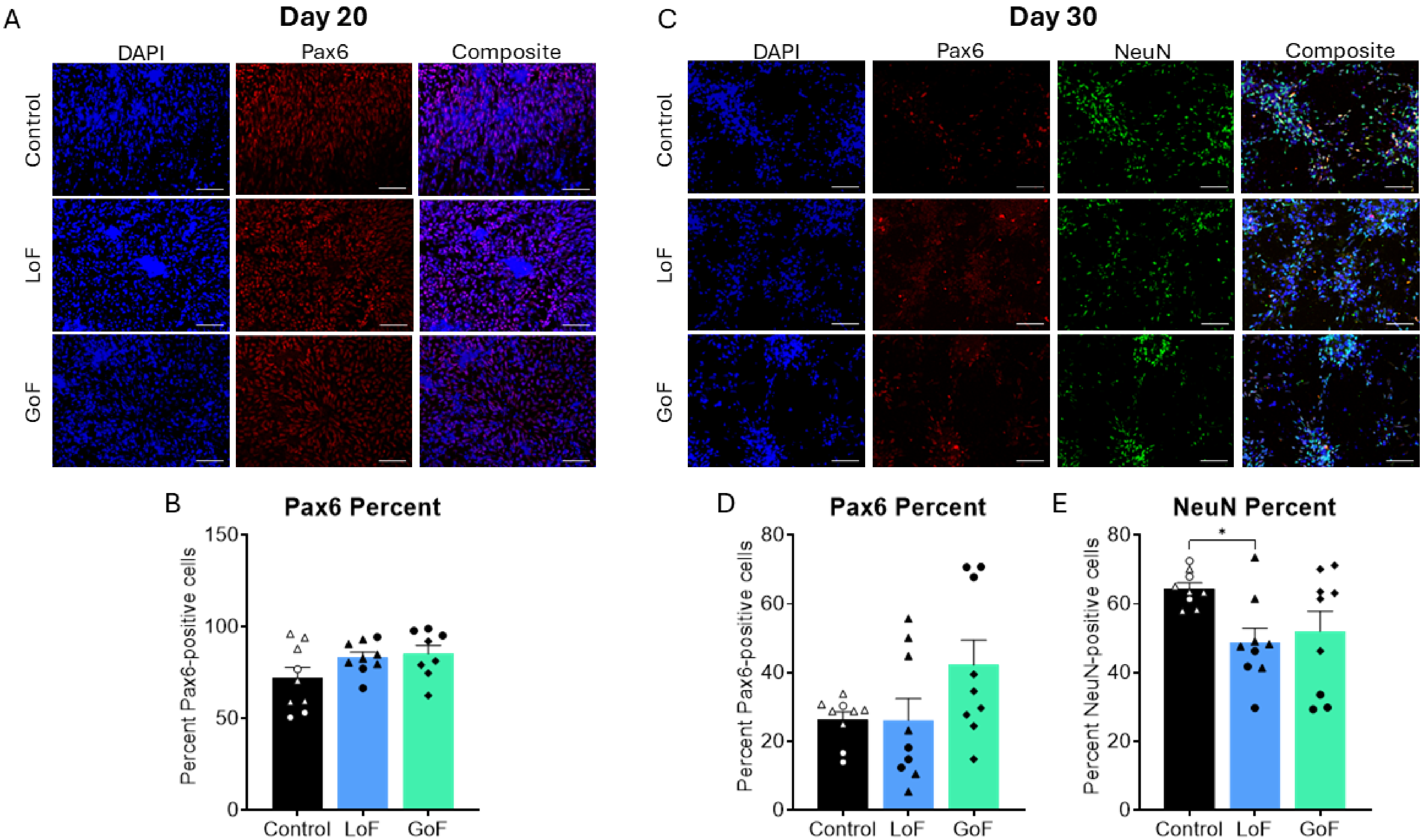
A
Day 20
DAPI
Pax6
Composite
Control
LoF
GoF
C
Day 30
DAPI
Pax6
NeuN
Composite
Control
LoF
GoF
B
Pax6 Percent
Percent Pax6-positive cells
150
100
50
0
Control
LoF
GoF
D
Pax6 Percent
Percent Pax6-positive cells
80
60
40
20
0
Control
LoF
GoF
E
NeuN Percent
Percent NeuN-positive cells
*
80
60
40
20
0
Control
LoF
GoF

Figure S4: Neuronal differentiation markers in CACNA1C LoF and GoF lines. A: Examples images of Pax6 staining at day 20 of neuronal differentiation in control, CACNA1C LoF and CACNA1C GoF lines. **B:** Quantification of the percentage of DAPI+ nuclei that colocalized with Pax6 at day 20. **C:** Examples images of Pax6 and NeuN staining at day 30 of neuronal differentiation in control, CACNA1C LoF and CACNA1C GoF lines. **D:** Quantification of the percentage of DAPI+ nuclei that colocalized with Pax6 at day 30. **E:** Quantification of the percentage of DAPI+ nuclei that colocalized with NeuN at day 30. All plots show mean ± SEM, individual replicates are overlayed as points. *$p < 0.05$, **$p < 0.01$, *** $p < 0.001$.

Supplemental References

1. Chambers SM, Fasano CA, Papapetrou EP, Tomishima M, Sadelain M, Studer L (2009): Highly efficient neural conversion of human ES and iPS cells by dual inhibition of SMAD signaling. *Nat Biotechnol* 27: 275–280.

2. Plumbly W, Brandon N, Deeb TZ, Hall J, Harwood AJ (2019): L-type voltage-gated calcium channel regulation of in vitro human cortical neuronal networks. *Sci Rep* 9: 1–12.

3. Wolstencroft J, Wicks F, Srinivasan R, Wynn S, Ford T, Baker K, *et al.* (2022): Neuropsychiatric risk in children with intellectual disability of genetic origin: IMAGINE, a UK national cohort study. *Lancet Psychiatry* 9: 715–724.

4. Alsaqati M, Heine VM, Harwood AJ (2020): Pharmacological intervention to restore connectivity deficits of neuronal networks derived from ASD patient iPSC with a TSC2 mutation. *Mol Autism* 11: 1–13.

5. Bader BM, Steder A, Klein AB, Frølund B, Schroeder OHU, Jensen AA (2017): Functional characterization of GABAA receptor-mediated modulation of cortical neuron network activity in microelectrode array recordings. *PLoS One* 12: e0186147.
